# Exploiting Bacterial Whole-Genome Sequencing Data for Evaluation of Diagnostic Assays: Campylobacter Species Identification as a Case Study

**DOI:** 10.1128/JCM.01522-16

**Published:** 2016-11-23

**Authors:** Melissa J. Jansen van Rensburg, Craig Swift, Alison J. Cody, Claire Jenkins, Martin C. J. Maiden

**Affiliations:** a Department of Zoology, University of Oxford, Oxford, United Kingdom; b NIHR Health Protection Research Unit in Gastrointestinal Infections, University of Oxford, Oxford, United Kingdom; c Gastrointestinal Bacteria Reference Unit, Public Health England, London, United Kingdom; Medical College of Wisconsin

## Abstract

The application of whole-genome sequencing (WGS) to problems in clinical microbiology has had a major impact on the field. Clinical laboratories are now using WGS for pathogen identification, antimicrobial susceptibility testing, and epidemiological typing. WGS data also represent a valuable resource for the development and evaluation of molecular diagnostic assays, which continue to play an important role in clinical microbiology. To demonstrate this application of WGS, this study used publicly available genomic data to evaluate a duplex real-time PCR (RT-PCR) assay that targets *mapA* and *ceuE* for the detection of Campylobacter jejuni and Campylobacter coli, leading global causes of bacterial gastroenteritis. *In silico* analyses of *mapA* and *ceuE* primer and probe sequences from 1,713 genetically diverse C. jejuni and C. coli genomes, supported by RT-PCR testing, indicated that the assay was robust, with 1,707 (99.7%) isolates correctly identified. The high specificity of the *mapA-ceuE* assay was the result of interspecies diversity and intraspecies conservation of the target genes in C. jejuni and C. coli. Rare instances of a lack of specificity among C. coli isolates were due to introgression in *mapA* or sequence diversity in *ceuE*. The results of this study illustrate how WGS can be exploited to evaluate molecular diagnostic assays by using publicly available data, online databases, and open-source software.

## INTRODUCTION

Accurate and timely diagnosis of infectious diseases is a cornerstone of clinical microbiology. Notwithstanding the ongoing importance of conventional culture in many settings, molecular diagnostics have markedly improved pathogen detection and identification (1). The most recent development in this area is the application of whole-genome sequencing (WGS) to problems in clinical microbiology (2–5). Although WGS is transforming the field, genomics and rapid molecular tests have complementary roles to play in diagnostic microbiology, particularly in resource-limited environments.

Since their introduction in the 1980s, nucleic acid amplification tests (NAATs), including multiplex assays that facilitate syndrome-driven diagnosis, have come to be widely used in bacteriology laboratories (1). In particular, multiplex NAATs are becoming increasingly popular for the identification of gastrointestinal pathogens, which include a wide range of viruses, bacteria, and parasites (6, 7). Many NAATs have, however, been designed by using representative nucleotide sequences from a limited number of isolates. During the pre-WGS era, the performance of NAATs could not be examined at the population level because the requisite large isolate collections were challenging to assemble and primer sequences were difficult to determine by Sanger sequencing.

The recent increase in WGS has generated an abundance of publicly available genomic data that have the potential to improve the development and evaluation of NAATs and other molecular diagnostics (8). At the time of writing of this work, growing numbers of assembled bacterial genomes were becoming available in public repositories, such as the NCBI (https://www.ncbi.nlm.nih.gov/genomes/MICROBES/microbial_taxtree.html); however, the majority of WGS data were available only as unassembled short reads. This limited their use to laboratories with bioinformatics expertise and resources. The PubMLST databases (http://pubmlst.org) address this issue by making large numbers of *de novo* assembled bacterial genomes publicly available through a web interface with analysis tools (9, 10). As of September 2016, the Ribosomal Multilocus Sequence Typing (rMLST) Database (http://pubmlst.org/rmlst/) (11) contained over 180,000 assembled bacterial genomes, which corresponded to more than 4,500 bacterial species. Similarly, an increasing number of species-specific PubMLST databases are also being populated with WGS data.

This study demonstrates how WGS data can be exploited to evaluate diagnostic assays. Campylobacter bacteria, a leading cause of bacterial gastroenteritis (12), were used as an exemplar. As Campylobacter bacteria are difficult to culture and identify, NAATs have become a popular tool for the diagnosis of campylobacteriosis (7, 13, 14); however, the extent to which existing assays are affected by the high levels of genetic diversity common among clinical isolates (15), or introgression, that is, the transfer of DNA between Campylobacter species (16–18), is unknown. The case study presented here is a duplex TaqMan real-time PCR (RT-PCR) for identification of Campylobacter jejuni and Campylobacter coli (19). Developed in the pre-WGS era using single gene sequences, the assay and variations thereof have been used for routine isolate identification to the species level (19–21); studies of Campylobacter isolates from humans (22–25), animals (26–35), and the environment (36); and outbreak investigations (37). For C. jejuni, the RT-PCR target is *mapA*, which encodes a putative outer membrane lipoprotein (38) shown to be immunogenic in chickens (39, 40). For C. coli, the target is *ceuE*, which encodes a periplasmic binding protein involved in iron scavenging (41). As *mapA* and *ceuE* are present in both organisms, the species specificity of the assay is contingent on the conservation of primer- and probe-binding sequences. Accordingly, the *mapA-ceuE* RT-PCR provided an opportunity to explore the utility of genomics and population genetics approaches for *in silico* evaluations of diagnostic assays.

## MATERIALS AND METHODS

### WGS data.

Best and colleagues (19) validated the *mapA-ceuE* assay by using clinical Campylobacter isolates from the United Kingdom. As Campylobacter genotypes circulating in Oxfordshire are representative of the United Kingdom (15, 25, 42, 43) and other high-income countries (http://pubmlst.org/campylobacter/), the Oxfordshire sentinel surveillance collection (15) was identified as an appropriate source of WGS data for this study. WGS data from 1,724 Campylobacter isolates were accessed via the Campylobacter jejuni/*coli* PubMLST database (http://pubmlst.org/campylobacter/). These Campylobacter bacteria comprised all of the single patient isolates recovered in Oxfordshire between June 2011 and June 2013.

### Genome annotation and data extraction.

The autotagger functionality within the PubMLST Bacterial Isolate Genome Sequence Database (BIGSdb) software (9) was used to identify *mapA* (PubMLST locus id CAMP0952), *ceuE* (CAMP1271), and the 7 multilocus sequence typing (MLST) (44, 45) and 52 rMLST (11) loci. Sequences with ≥98% identity and ≥98% alignment with existing alleles were annotated automatically. Using curation tools available in PubMLST, predicted sequences with 70 to 98% identity to existing alleles were aligned at the nucleotide and amino acid sequence levels with the closest match in the database. Following visual inspection of the alignments, complete coding sequences were added to the database. Those with internal stop codons were “flagged,” that is, highlighted in the database, and marked as “visually checked.” Allelic data and corresponding nucleotide sequences, MLST-defined sequence types (STs), clonal complexes, and ribosomal STs (rSTs) were exported from the database by using the BIGSdb data export plugin (9).

### Isolate diversity and species identification.

The allelic diversity of the MLST and rMLST data was determined by using the bias-corrected version of Simpson's index of diversity (*D*) (46, 47) with 95% confidence intervals (CIs) (48). Possible values of *D* ranged from 0 (no diversity) to 1 (maximum diversity). The distribution of MLST clonal complexes was compared to that observed for 3,349 human disease isolates recovered in Oxfordshire between 2003 and 2009 (42). Study isolates were assigned to species groups by using rMLST (11). For this analysis, concatenated nucleotide sequences of unique rSTs (∼20,780 bp) were aligned with MAFFT version 7.037b (49). The memory requirements for maximum-likelihood (ML) analysis of the study data set exceeded that of a standard installation of MEGA version 5.05; therefore, an ML phylogeny was generated on a Linux server with MEGA-CC version 7.0 (50) by using the general time-reversible model with gamma-distributed rates plus invariant sites with 500 bootstrap replicates (51). This analysis required knowledge of the command line and took 9 days. As usability and computational speed were considered important factors in this study, the ML phylogeny was compared to a neighbor-joining tree (52) reconstructed in MEGA version 5.05 (51) with the Kimura two-parameter model (53) by using 1,000 bootstrap replicates. At the population level, C. coli segregates into three clades (54), and additional rMLST analyses were carried out to resolve the assignment of a subset of isolates to these groups. The approach described above was used to compare rSTs of interest to a reference set of 15 C. coli genomes representative of the three clades, with the ML phylogeny generated with the Tamura-Nei model with gamma-distributed rates plus invariant sites with 500 bootstrap replicates (see Table S2 in the supplemental material) (16, 55, 56).

### *In silico* assay evaluation.

Nucleotide sequence alignments of unique *mapA* and *ceuE* alleles were generated as for the rMLST phylogeny, and regions corresponding to the forward primer, probe, and reverse primer (19) were extracted. Primer and probe nucleotide sequence fragments were aligned and concatenated, and unique combinations were assigned allele numbers in the order of discovery.

### RT-PCR confirmation of *in silico* evaluation results.

Archived genomic DNA and bacterial cultures were available for the study isolates (15), which facilitated RT-PCR confirmation of the *in silico* evaluation results. Representative isolates (*n* = 124) were chosen for RT-PCR such that each unique *mapA* and *ceuE* forward primer, probe, and reverse primer combination was tested at least once, with the subset also representative of the genetic diversity of the study data set. For isolates with insufficient archived genomic DNA (*n* = 5), glycerol stocks of single-colony cultures were inoculated onto Columbia agar with horse blood (Oxoid Ltd., Basingstoke, United Kingdom) and incubated in a microaerobic atmosphere at 42°C for 48 h. Boiled cell lysates were prepared from single colonies as previously described (19). RT-PCR was carried out according to the method of Best et al. (19), and positive results were defined as those with cycle threshold (*C_T_*) values ranging from 12 to 30.

### Genetic diversity, introgression, and selection in RT-PCR targets.

Individual *mapA* and *ceuE* nucleotide sequence alignments and gene phylogenies were generated as described for rMLST. The *mapA* ML phylogeny was constructed with the Tamura three-parameter model with gamma distributed rates with 500 bootstrap replicates, and the same parameters were used for *ceuE*, with the addition of invariant sites. Nucleotide sequences were translated with MEGA version 5.05 (51), and allele numbers were assigned to unique protein sequences. STRUCTURE (57), a Bayesian clustering algorithm, was used to characterize introgression in *mapA* and *ceuE* as previously described (17, 18). Isolates were probabilistically assigned to species with the linkage model, which adjusts for linkage disequilibrium between nucleotides (58). The model was run with default settings for 10,000 burn-in iterations and 10,000 additional iterations, assuming a population number (*k*) of 2. Putative mosaic alleles were identified as those with a ≤0.75 probability of belonging to either C. jejuni or C. coli (18). Site-by-site frequencies generated by STRUCTURE were used to identify nucleotide sequence fragments with different ancestries in putative mosaic alleles (18, 58). After putative recombinant alleles were excluded, within- and between-group *p* distances were calculated for C. jejuni- and C. coli-specific gene and protein sequences with DnaSP version 5.10 (59). Species-specific synonymous and nonsynonymous substitution rates (*dN*/*dS*) were calculated for *mapA* and *ceuE* alleles encoding full-length protein sequences with SNAP version 2.1.1 (www.hiv.lanl.gov) (60).

## RESULTS

### Isolate diversity and species identification.

**FIG 1:**
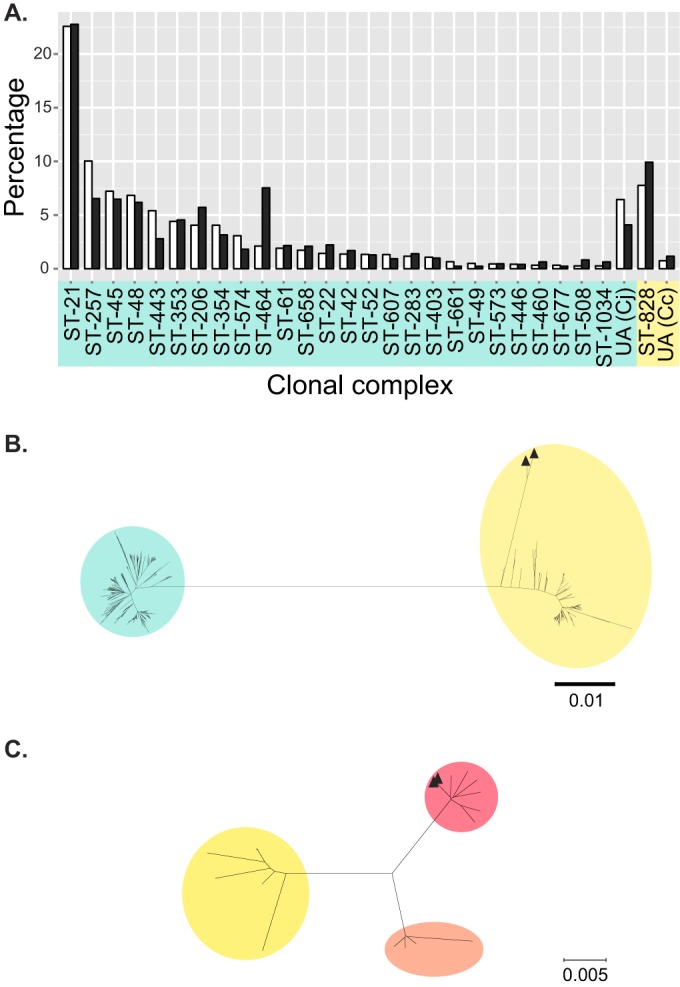
Genetic diversity and species identification of 1,713 Campylobacter genomes from Oxfordshire human disease isolates (2011 to 2013). (A) Frequency distribution of major clonal complexes (*n* = ≥10) among 3,349 Campylobacter isolates from human disease cases in Oxfordshire (2003 to 2009) typed by MLST (white) (42) and the 1,713 genomes included in this study (black). UA, STs unassigned to a clonal complex; Cj, C. jejuni (blue); Cc, C. coli (yellow). (B) Neighbor-joining tree based on concatenated nucleotide sequences of unique rMLST profiles (*n* = 597) identified among the study isolates. ▲, putative clade 3 C. coli. (C) Neighbor-joining tree based on concatenated nucleotide sequences of rMLST profiles of 15 representative isolates belonging to C. coli clades 1 (yellow), 2 (orange), and 3 (pink) and three putative clade 3 isolates identified in this study (▲).

Complete nucleotide sequences of *mapA* and *ceuE* and the MLST and rMLST loci were obtained from 1,713/1,724 (99.4%) isolates (see Table S1 in the supplemental material), excluding those with: incomplete MLST and/or rMLST profiles (*n* = 8), misassembled *mapA* or *ceuE* sequences (*n* = 1), or multiple alleles at any of the rMLST loci (*n* = 2), which is an indicator that a mixed culture may have been sequenced. The isolates included can be accessed via the Campylobacter jejuni/*coli* PubMLST isolate database and are grouped in the *mapA-ceuE* evaluation project. The collection comprised 293 STs (*D* = 0.974 [95% CI, 0.972 to 0.976]) and 597 rSTs (*D* = 0.989 [95% CI, 0.988 to 0.991]). The STs were assigned to 33 clonal complexes, with proportions similar to those observed previously in Oxfordshire (Fig. 1A) (15, 42). Species designations were inferred from the ML and neighbor-joining rMLST phylogenies (11), which aggregated rSTs into identical species groups. As the same was true for all paired phylogenies, only the neighbor-joining trees are presented here. C. jejuni accounted for 1,521 (88.8%) isolates, and C. coli accounted for the remaining 192 (11.2%) (Fig. 1B). Two C. coli rSTs, rST398 (*n* = 2) and rST4701 (*n* = 1), were distinct from the other C. coli sequences and occurred at the tip of a long branch (Fig. 1B). Further rMLST analyses indicated that these isolates belonged to C. coli clade 3 (Fig. 1C).

### *In silico* assay evaluation.

There were 72 *mapA* alleles of 645 bp and 126 *ceuE* alleles of 990 to 994 bp represented in the isolate collection. Differences in *ceuE* allele lengths were due mainly to variation in three homopolymeric tracts, which resulted in internal stop codons in 11 C. jejuni-specific alleles (*n* = 20) (Table 1). These alleles were flagged and marked as visually checked in the database. To evaluate assay specificity, primer- and probe-binding sequences were extracted from *mapA* and *ceuE* and analyzed in detail.

**TABLE 1:** Details of *ceuE* alleles with internal stop codons identified among C. jejuni isolates

Polymorphism	Nucleotide position	Putative effect on protein	PubMLST gene allele	ST/CC^*a*^/rST (*n*)
T(8 → 7)	34	Truncation	288	5756/UA^*b*^/263 (1)
			290	2844/ST460/325 (2)
			291	48/ST48/106 (2), 48/ST48/98 (1), 48/ST48/99 (1), 520/ST21/377 (1)
			293	2274/UA/123 (1)
			294	443/ST443/221 (1)
			296	5707/UA/3509 (1)
			298	1932/ST460/4596 (2)
			300	464/ST464/7025 (1)
T(8 → 9)	34	Truncation	295	21/ST21/538 (1)
A(5 → 4)	202	Truncation	289	47/ST21/510 (3), 3633/ST21/510 (1)
T(6 → 5)	483	Truncation	292	53/ST21/460 (1)
Deletion (C)	675	Truncation	297	257/ST257/186 (2)

a CC, MLST-defined clonal complex.

b UA, ST not assigned to a clonal complex.

### *mapA*.

**FIG 2:**
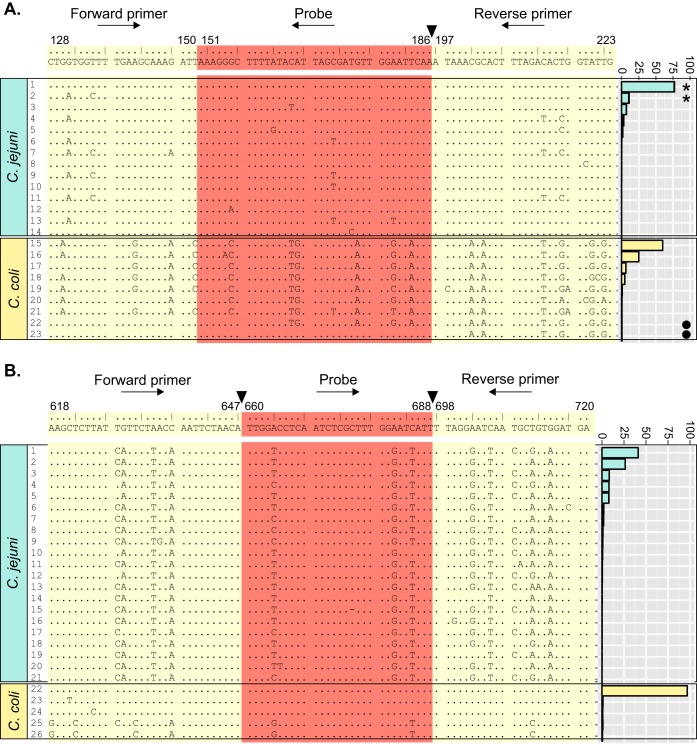
Genetic diversity of *mapA* (A) and *ceuE* (B) primer and probe sequences. Published primer (cream) and probe (orange) sequences (19) are shown above concatenated nucleotide sequence alignments of unique combinations identified in genomes from campylobacteriosis cases in Oxfordshire (2011 to 2013). Dots represent conserved nucleotides. Numbers above the published primers and probes indicate nucleotide positions relative to the complete gene, with breaks between regions marked (▼). Adjacent histograms indicate frequencies of combinations in C. jejuni (blue) and C. coli (yellow). *, complete C. jejuni-specific combination detected in a single C. coli isolate; ●, composite nonspecific combination detected in a single C. coli isolate.

Twenty-three unique *mapA* primer-and-probe combinations were identified among the study isolates. Twelve were present only in isolates designated C. jejuni, nine were in C. coli, and two were in both species. Two distinct groups, consistent with microbiological species, were evident from the nucleotide sequence alignment of these unique combinations (Fig. 2A). Primer and probe sequences were conserved among C. jejuni isolates. The predominant primer-and-probe combination was detected in 1,166 (76.7%) isolates and was identical to the published sequences (19). Sequence variation among divergent C. jejuni combinations was limited to between one and five polymorphisms across the three regions. C. coli sequences were also conserved but were divergent from C. jejuni combinations, differing from the published sequences (19) at up to 18 sites (Fig. 2A); however, four C. coli isolates carried nonspecific primer-and-probe combinations (Table 2). Two combinations, each present in a single C. coli isolate, corresponded to predominant C. jejuni-specific alleles 1 and 2 (Fig. 2A). The remaining two nonspecific combinations were composites of C. coli and C. jejuni sequences (Table 2; Fig. 2A).

**TABLE 2:** Details of C. coli isolates with atypical *mapA*/*ceuE* primer and probe sequences

Atypical target and isolate	ST (CC)^*a*^	Gene allele/primer and probe combination (RT-PCR result)^*b*^		*mapA*/*ceuE* RT-PCR result
		*mapA*	*ceuE*	
*mapA*				
OXC7352	6973 (ST1150)	20/2 (+)	136/22 (+)	Mixed
OXC6987	825 (ST828)	88/1 (+)	3/22 (+)	Mixed
OXC6395	1487 (ST1150)	19/22^*d*^ (−)	17/22 (+)	C. coli
OXC7615	6760 (UA^*c*^)	111/23^*e*^ (late +)	139/22 (+)	Inconclusive
*ceuE*				
OXC7241	6698 (UA)	96/19 (−)	153/25 (late +)	Inconclusive
OXC7243	6698 (UA)	96/19 (−)	153/25 (late +)	Inconclusive
OXC7653	6975 (UA)	114/21 (−)	183/26 (late +)	Inconclusive

a CC, clonal complex.

b +, target detected; −, target not detected; late +, target detected after cycle 30.

c UA, ST not assigned to a clonal complex.

d Forward primer *C*. jejuni specific, probe and reverse primer C. coli specific.

e Forward primer and probe *C*. jejuni specific, reverse primer C. coli specific.

### *ceuE*.

The 26 *ceuE* primer-and-probe combinations identified among the study isolates were all species specific. Twenty-one were present only in C. jejuni, and five were present only in C. coli. Primer-and-probe combinations were also stratified by species at the nucleotide sequence level (Fig. 2B). C. coli sequences were highly conserved, with 186 (96.9%) isolates identical to the published primer and probe sequences (19). Nucleotide variation was limited to between one and eight polymorphisms per primer-and-probe combination, the majority of which occurred in alleles 25 (*n* = 2) and 26 (*n* = 1), which were present in the clade 3 C. coli isolates (Table 2; Fig. 2B). In contrast, C. jejuni combinations were divergent from the published sequences (19), containing between 10 and 13 nucleotide sequence differences across the three regions. C. jejuni isolates were also more evenly distributed across primer and probe sequences, with eight combinations accounting for 96.3% of the isolates, in contrast to a single combination accounting for 96.9% of the C. coli isolates (Fig. 2B).

### Predicted assay performance and RT-PCR confirmation.

Predicted species designations based on the results of the *in silico* evaluation were consistent with rMLST species assignments for 1,707/1,713 (99.7%) isolates, corresponding to 1,521 (100%) of the C. jejuni and 186 (96.9%) of the C. coli isolates. These results were confirmed by RT-PCR testing of 124 representative isolates. C. coli isolates with complete C. jejuni-specific *mapA* primer and probe sequences were *mapA* positive/*ceuE* positive (Table 2). RT-PCR results for the C. coli isolate carrying C. jejuni-specific *mapA* forward primer and probe sequences and the three clade 3 C. coli isolates were inconclusive, as the *C_T_* values for *mapA* and *ceuE*, respectively, ranged from 32 to 37, exceeding the assay cutoff of 30 (19) (Table 2; see Table S3 in the supplemental material). Although this study was not designed to quantify the effects of primer and probe mismatches on target detection, there was a correlation between the number of polymorphisms and *C_T_* values (see Table S3).

### Introgression, diversity, and selection in RT-PCR targets.

**FIG 3:**
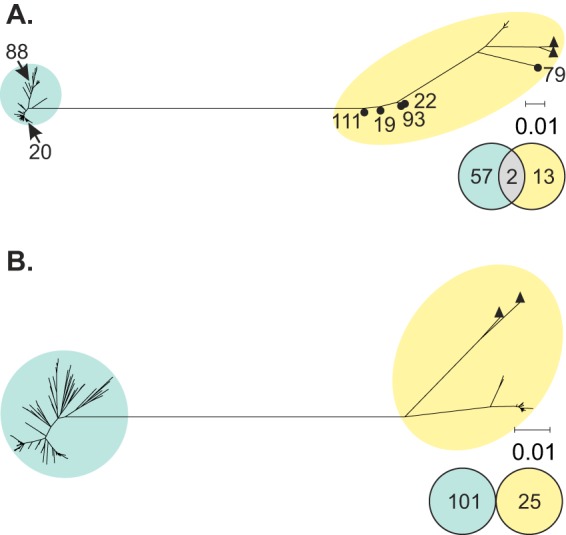
Neighbor-joining trees showing relationships among 72 *mapA* (A) and 126 *ceuE* (B) unique gene sequences from Campylobacter genomes from cases of human disease in Oxfordshire (2011 to 2013). Adjacent Venn diagrams indicate the numbers of species-specific and shared alleles. X →, C. jejuni-specific allele detected in C. coli, where X is the allele number; ●, putative introgressed alleles; ▲, putative clade 3 C. coli alleles. C. jejuni, blue; C. coli, yellow; shared alleles, gray.

Additional analyses were carried out at the whole-gene level to explore the impact of introgression, diversity, and selection on assay specificity. Individual gene phylogenies confirmed that *mapA* and *ceuE* alleles were species specific (Fig. 3), with the exception of *mapA* alleles 20 and 88, which were present in the *mapA*-positive/*ceuE*-positive C. coli isolates (Fig. 3A; Table 2). Clade 3 C. coli *mapA* and *ceuE* alleles clustered with the other C. coli sequences; however, they were distinct from clade 1 sequences and were at the end of a long branch in both phylogenies, indicative of genetic divergence (Fig. 3). Also noteworthy were five C. coli alleles that occupied intermediate positions on the *mapA* phylogeny (Fig. 3A). Taken together with the interspecies transfer of alleles 20 and 88, these findings indicated introgression in *mapA*, which supported the results of the *in silico* evaluation.

**FIG 4:**
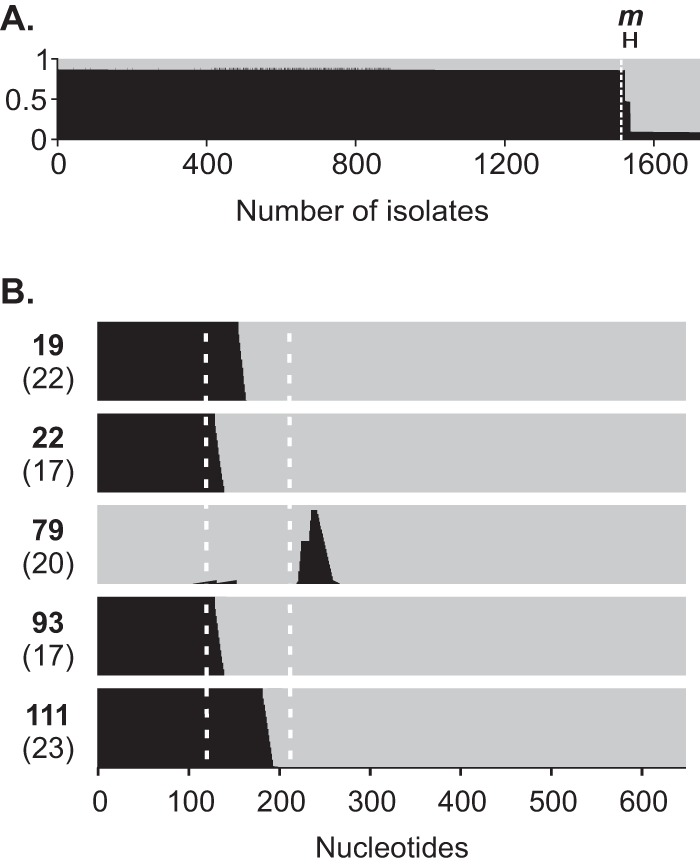
Characterization of introgression in *mapA* with STRUCTURE. (A) Probabilistic assignment of study isolates to species based on analysis of *mapA* nucleotide sequences with the linkage model. Putative mosaic sequences (delineated by a horizontal bar and marked “*m*”) were identified as those with a ≤0.75 probability of belonging to either C. jejuni or C. coli. Each isolate is represented by a vertical line, with shading indicative of the proportion attributed to C. jejuni (black) or C. coli (gray) ancestry. The dashed white line indicates the species boundary as determined by rMLST. (B) Recombination breakpoints in putative mosaic alleles were inferred by using site-by-site nucleotide ancestries generated by STRUCTURE. Bar plots represent individual putative mosaic sequences, with whole-gene allele numbers and corresponding primer-and-probe combinations shown in bold and in parentheses, respectively. Vertical lines represent individual nucleotides with shading indicative of ancestry as in panel A. Dashed white lines demarcate the region amplified by *mapA* primers (19).

Drawing on population genetics approaches, introgression in the RT-PCR target genes was formally characterized with STRUCTURE, with mixed ancestry detected only in the *mapA* gene of 17 (8.9%) C. coli isolates. In addition to the two previously identified complete gene transfers, five putative mosaic alleles were detected (*n* = 15) (Fig. 4A). All imported DNA was identical to the predominant C. jejuni sequence. Recombination breakpoints occurred within the amplified region in four introgressed alleles, of which alleles 19 and 111 corresponded to composite primer and probe sequences (Fig. 4B). Alleles 22 (*n* = 11) and 93 (*n* = 1) were not identified as introgressed sequences during the *in silico* evaluation because the putative breakpoints occurred at the 5′ end of the forward primer (Fig. 4B).

For both *mapA* and *ceuE*, between-species *p* distances were at least an order of magnitude greater than those within species, and lower levels of diversity were observed for the targeted species (Table 3). Analyses carried out at the allele level indicated that gene and protein diversity was primarily due to an abundance of rare alleles (see Fig. S1 in the supplemental material). The average *dN*/*dS* ratios for *mapA* and *ceuE* were <1 (0.077 to 0.14) in C. jejuni and C. coli, consistent with both genes being under stabilizing selection; however, the distribution of synonymous and nonsynonymous substitutions suggested species-specific differences in *mapA* and *ceuE* evolution (see Fig. S2 in the supplemental material).

**TABLE 3:** Intra- and interspecies diversity of *mapA* and *ceuE* gene and protein sequences^*a*^

Parameter and species	*p* distance	
	*mapA*	*ceuE*
Gene sequence diversity		
C. jejuni	0.014	0.018
C. coli	0.023	0.005
C. jejuni/C. coli	0.232	0.13
Protein sequence diversity		
C. jejuni	0.009	0.015
C. coli	0.020	0.009
C. jejuni/C. coli	0.232	0.089

a Putative recombinant sequences and alleles encoding truncated peptide sequences were excluded.

## DISCUSSION

This study demonstrates how bacterial WGS data can be used indirectly to support diagnostic laboratory activities. *In silico* analyses of primer and probe sequences, in conjunction with RT-PCR, confirmed that the *mapA-ceuE* assay was robust. Overall, 1,707 (99.7%) isolates were correctly identified, which was similar to the 97.7% level of accuracy reported during test validation by conventional approaches (19). Assay specificity was attributable to a combination of interspecies diversity and marked intraspecies conservation within primer- and probe-binding regions, in addition to overrepresentation of sequences identical to published primers and probes (Fig. 2).

Experimental evidence suggests that the products of *mapA* and *ceuE* play roles in colonization (39) and iron acquisition (61), respectively, in Campylobacter species. Whole-gene analyses indicated that intraspecies diversity was low at the gene and protein levels and that both targets were under stabilizing selection (Table 3). One possible explanation for these findings is that *mapA* and *ceuE* encode essential cellular components in C. jejuni and C. coli. This is supported by the results of experimental studies in which *mapA* and *ceuE* mutants showed a reduced potential for chicken colonization (39, 62). Species-specific differences in the distribution of synonymous and nonsynonymous substitutions in *mapA* and *ceuE* suggest divergent evolution in C. jejuni and C. coli postspeciation (see Fig. S2 in the supplemental material), perhaps because of host niche differences (35, 54, 63–65). Interestingly, the predicted protein sequences of 11 C. jejuni-specific *ceuE* alleles (*n* = 20) were truncated because of variation in three homopolymeric tracts, one of which occurred at the 5′ end of the gene (Table 1). Sequencing of NCTC 11168 demonstrated that homopolymeric tracts are common in the C. jejuni genome, with multiple variants detected in clones that were otherwise indistinguishable. These hypervariable sequences regulate gene expression through phase variation (66). It is possible that *ceuE* is also phase variable, although confirmation of homopolymeric tract lengths was beyond the scope of this study.

Although a small proportion of Campylobacter isolates could not be identified to the species level with the *mapA-ceuE* assay, both targets were universally present and no isolates were incorrectly identified. Only six (3.1%) C. coli isolates could not be identified, including two *mapA*-positive/*ceuE*-positive isolates and four isolates with inconclusive results due to late detection of *mapA* (*n* = 1) or *ceuE* (*n* = 3) (*C_T_* values, 32 to 37) (Table 2; see Table S3 in the supplemental material). Introgression by horizontal gene transfer (HGT) was the underlying cause of *mapA* detection among C. coli isolates (Fig. 4). HGT can result in the transfer of complete genes (whole-allele replacement) or the generation of mosaic alleles. While whole-allele replacements were relatively uncommon (1%), they accounted for the *mapA*-positive/*ceuE*-positive C. coli isolates. Mosaic alleles were more prevalent (7.8%) but resulted in only one inconclusive RT-PCR result. The apparent lack of introgression in *ceuE* may be due to functional and combinatorial epistasis, as the gene is part of the *ceuBCDE* operon, the products of which form an inner membrane ABC transporter system (41). Those isolates that could not be conclusively identified because of late detection of *ceuE* corresponded to clade 3 C. coli. While clade 1 C. coli strains account for the majority of human disease and agricultural isolates, strains belonging to clades 2 and 3 are generally from environmental sources (54). Phylogenetic analyses showed that clade 3 *ceuE* sequences were divergent from other C. coli-specific alleles (Fig. 3B). An accumulation of mutations in the forward primer region reduced the amplification efficiency (Fig. 2B; see Table S3), indicating a lack of assay specificity for clade 3 C. coli.

Taken together, the results of the *in silico* evaluation showed that the *mapA-ceuE* RT-PCR assay reliably identifies C. jejuni and C. coli, while whole-gene analyses provided insights into underlying reasons for the specificity of the assay. The value of these findings also extends to other diagnostic assays that use *mapA* or *ceuE* as a target (20, 21, 67–70). In the United Kingdom and other high-income countries, the impact of RT-PCR failures observed in this study would be limited because (i) C. coli accounts for a small proportion of human campylobacteriosis cases (12), (ii) the RT-PCR result was unaffected for the majority of isolates with introgressed *mapA* alleles, and (iii) clade 2 and 3 C. coli strains rarely cause human disease (54). Given that signals of host association are more marked than geographic signals (35), it is likely that the assay will perform well in other regions where food animals similar to those consumed in the United Kingdom are consumed; however, laboratories in regions with discernible differences in Campylobacter epidemiology should exercise caution and validate the assay prior to use.

The *in silico* approach to assay evaluation used here could be extended to other NAATs or molecular diagnostic tests. Compared to Sanger sequencing, WGS represents an attractive alternative for studying primer sequences, particularly those with mismatches that adversely affect amplification efficiency. The approach outlined in this study could be used to evaluate existing assays, or it could be applied in conjunction with primer design software during assay development, requiring only a personal computer with internet access and publicly available software.

## Supplementary Material

Supplemental material

## References

[1] Buchan BW, Ledeboer NA 2014 Emerging technologies for the clinical microbiology laboratory. Clin Microbiol Rev 27:783–822. doi:10.1128/CMR.00003-14. 25278575 PMC4187641

[2] Didelot X, Bowden R, Wilson DJ, Peto TEA, Crook DW 2012 Transforming clinical microbiology with bacterial genome sequencing. Nat Rev Genet 13:601–612. doi:10.1038/nrg3226. 22868263 PMC5049685

[3] Köser CU, Ellington MJ, Cartwright EJ, Gillespie SH, Brown NM, Farrington M, Holden MT, Dougan G, Bentley SD, Parkhill J, Peacock SJ 2012 Routine use of microbial whole genome sequencing in diagnostic and public health microbiology. PLoS Pathog 8:e1002824. doi:10.1371/journal.ppat.1002824. 22876174 PMC3410874

[4] Köser CU, Ellington MJ, Peacock SJ 2014 Whole-genome sequencing to control antimicrobial resistance. Trends Genet 30:401–407. doi:10.1016/j.tig.2014.07.003. 25096945 PMC4156311

[5] Robinson ER, Walker TM, Pallen MJ 2013 Genomics and outbreak investigation: from sequence to consequence. Genome Med 5:36. doi:10.1186/gm440. 23673226 PMC3706975

[6] Platts-Mills JA, Liu J, Houpt ER 2013 New concepts in diagnostics for infectious diarrhea. Mucosal Immunol 6:876–885. doi:10.1038/mi.2013.50. 23881355

[7] Zhang H, Morrison S, Tang YW 2015 Multiplex polymerase chain reaction tests for detection of pathogens associated with gastroenteritis. Clin Lab Med 35:461–486. doi:10.1016/j.cll.2015.02.006. 26004652 PMC5002946

[8] Fournier PE, Dubourg G, Raoult D 2014 Clinical detection and characterization of bacterial pathogens in the genomics era. Genome Med 6:114. doi:10.1186/s13073-014-0114-2. 25593594 PMC4295418

[9] Jolley KA, Maiden MC 2010 BIGSdb: scalable analysis of bacterial genome variation at the population level. BMC Bioinformatics 11:595. doi:10.1186/1471-2105-11-595. 21143983 PMC3004885

[10] Maiden MC, Jansen van Rensburg MJ, Bray JE, Earle SG, Ford SA, Jolley KA, McCarthy ND 2013 MLST revisited: the gene-by-gene approach to bacterial genomics. Nat Rev Microbiol 11:728–736. doi:10.1038/nrmicro3093. 23979428 PMC3980634

[11] Jolley KA, Bliss CM, Bennett JS, Bratcher HB, Brehony CM, Colles FM, Wimalarathna HM, Harrison OB, Sheppard SK, Cody AJ, Maiden MC 2012 Ribosomal multi-locus sequence typing: universal characterization of bacteria from domain to strain. Microbiology 158:1005–1015. doi:10.1099/mic.0.055459-0. 22282518 PMC3492749

[12] Kaakoush NO, Castano-Rodriguez N, Mitchell HM, Man SM 2015 Global epidemiology of Campylobacter infection. Clin Microbiol Rev 28:687–720. doi:10.1128/CMR.00006-15. 26062576 PMC4462680

[13] Fitzgerald C 2015 Campylobacter. Clin Lab Med 35:289–298. doi:10.1016/j.cll.2015.03.001. 26004643

[14] On SL, Jordan PJ 2003 Evaluation of 11 PCR assays for species-level identification of Campylobacter jejuni and Campylobacter coli. J Clin Microbiol 41:330–336. doi:10.1128/JCM.41.1.330-336.2003. 12517869 PMC149560

[15] Cody AJ, McCarthy ND, Jansen van Rensburg M, Isinkaye T, Bentley S, Parkhill J, Dingle KE, Bowler IC, Jolley KA, Maiden MC 2013 Real-time genomic epidemiology of human Campylobacter isolates using whole-genome multilocus sequence typing. J Clin Microbiol 51:2526–2534. doi:10.1128/JCM.00066-13. 23698529 PMC3719633

[16] Sheppard SK, Didelot X, Jolley KA, Darling AE, Pascoe B, Meric G, Kelly DJ, Cody A, Colles FM, Strachan NJ, Ogden ID, Forbes K, French NP, Carter P, Miller WG, McCarthy ND, Owen R, Litrup E, Egholm M, Affourtit JP, Bentley SD, Parkhill J, Maiden MC, Falush D 2013 Progressive genome-wide introgression in agricultural Campylobacter coli. Mol Ecol 22:1051–1064. doi:10.1111/mec.12162. 23279096 PMC3749442

[17] Sheppard SK, McCarthy ND, Falush D, Maiden MC 2008 Convergence of Campylobacter species: implications for bacterial evolution. Science 320:237–239. doi:10.1126/science.1155532. 18403712

[18] Sheppard SK, McCarthy ND, Jolley KA, Maiden MCJ 2011 Introgression in the genus Campylobacter: generation and spread of mosaic alleles. Microbiology 157:1066–1074. doi:10.1099/mic.0.045153-0. 21212120 PMC3139442

[19] Best EL, Powell NB, Swift C, Grant KA, Frost JA 2003 Applicability of a rapid duplex real-time PCR assay for speciation of Campylobacter jejuni and Campylobacter coli directly from culture plates. FEMS Microbiol Lett 229:237–241. doi:10.1016/S0378-1097(03)00845-0. 14680705

[20] Schuurman T, de Boer RF, van Zanten E, van Slochteren KR, Scheper HR, Dijk-Alberts BG, Moller AV, Kooistra-Smid AM 2007 Feasibility of a molecular screening method for detection of Salmonella enterica and Campylobacter jejuni in a routine community-based clinical microbiology laboratory. J Clin Microbiol 45:3692–3700. doi:10.1128/JCM.00896-07. 17804656 PMC2168500

[21] Van Lint P, De Witte E, De Henau H, De Muynck A, Verstraeten L, Van Herendael B, Weekx S 2015 Evaluation of a real-time multiplex PCR for the simultaneous detection of Campylobacter jejuni, Salmonella spp., Shigella spp/EIEC, and Yersinia enterocolitica in fecal samples. Eur J Clin Microbiol 34:535–542. doi:10.1007/s10096-014-2257-x. 25326870

[22] Amar CF, East CL, Gray J, Iturriza-Gomara M, Maclure EA, McLauchlin J 2007 Detection by PCR of eight groups of enteric pathogens in 4,627 faecal samples: re-examination of the English case-control Infectious Intestinal Disease Study (1993–1996). Eur J Clin Microbiol 26:311–323. doi:10.1007/s10096-007-0290-8. 17447091

[23] Mason J, Iturriza-Gomara M, O'Brien SJ, Ngwira BM, Dove W, Maiden MC, Cunliffe NA 2013 Campylobacter infection in children in Malawi is common and is frequently associated with enteric virus co-infections. PLoS One 8:e59663. doi:10.1371/journal.pone.0059663. 23555739 PMC3608717

[24] O'Brien SJ, Rait G, Hunter PR, Gray JJ, Bolton FJ, Tompkins DS, McLauchlin J, Letley LH, Adak GK, Cowden JM, Evans MR, Neal KR, Smith GE, Smyth B, Tam CC, Rodrigues LC 2010 Methods for determining disease burden and calibrating national surveillance data in the United Kingdom: the second study of infectious intestinal disease in the community (IID2 study). BMC Med Res Methodol 10:39. doi:10.1186/1471-2288-10-39. 20444246 PMC2886083

[25] Sopwith W, Birtles A, Matthews M, Fox A, Gee S, Painter M, Regan M, Syed Q, Bolton E 2006 Campylobacter jejuni multilocus sequence types in humans, northwest England, 2003–2004. Emerg Infect Dis 12:1500–1507. doi:10.3201/eid1210.060048. 17176563 PMC3290937

[26] Bull SA, Allen VM, Domingue G, Jorgensen F, Frost JA, Ure R, Whyte R, Tinker D, Corry JEL, Gillard-King J, Humphrey TJ 2006 Sources of Campylobacter spp. colonizing housed broiler flocks during rearing. Appl Environ Microbiol 72:645–652. doi:10.1128/AEM.72.1.645-652.2006. 16391102 PMC1352183

[27] Griggs DJ, Johnson MM, Frost JA, Humphrey T, Jorgensen F, Piddock LJ 2005 Incidence and mechanism of ciprofloxacin resistance in Campylobacter spp. isolated from commercial poultry flocks in the United Kingdom before, during, and after fluoroquinolone treatment. Antimicrob Agents Chemother 49:699–707. doi:10.1128/AAC.49.2.699-707.2005. 15673754 PMC547197

[28] Humphrey TJ, Jorgensen F, Frost JA, Wadda H, Domingue G, Elviss NC, Griggs DJ, Piddock LJ 2005 Prevalence and subtypes of ciprofloxacin-resistant Campylobacter spp. in commercial poultry flocks before, during, and after treatment with fluoroquinolones. Antimicrob Agents Chemother 49:690–698. doi:10.1128/AAC.49.2.690-698.2005. 15673753 PMC547194

[29] Kalupahana RS, Kottawatta KS, Kanankege KS, van Bergen MA, Abeynayake P, Wagenaar JA 2013 Colonization of Campylobacter spp. in broiler chickens and laying hens reared in tropical climates with low-biosecurity housing. Appl Environ Microbiol 79:393–395. doi:10.1128/AEM.02269-12. 23087035 PMC3536106

[30] Kwan PS, Birtles A, Bolton FJ, French NP, Robinson SE, Newbold LS, Upton M, Fox AJ 2008 Longitudinal study of the molecular epidemiology of Campylobacter jejuni in cattle on dairy farms. Appl Environ Microbiol 74:3626–3633. doi:10.1128/AEM.01669-07. 18424539 PMC2446552

[31] Rapp D, Ross CM, Pleydell EJ, Muirhead RW 2012 Differences in the fecal concentrations and genetic diversities of Campylobacter jejuni populations among individual cows in two dairy herds. Appl Environ Microbiol 78:7564–7571. doi:10.1128/AEM.01783-12. 22904055 PMC3485710

[32] Ridley A, Morris V, Gittins J, Cawthraw S, Harris J, Edge S, Allen V 2011 Potential sources of Campylobacter infection on chicken farms: contamination and control of broiler-harvesting equipment, vehicles and personnel. J Appl Microbiol 111:233–244. doi:10.1111/j.1365-2672.2011.05038.x. 21535329

[33] Ridley AM, Allen VM, Sharma M, Harris JA, Newell DG 2008 Real-time PCR approach for detection of environmental sources of Campylobacter strains colonizing broiler flocks. Appl Environ Microbiol 74:2492–2504. doi:10.1128/AEM.01242-07. 18203857 PMC2293161

[34] Ridley AM, Morris VK, Cawthraw SA, Ellis-Iversen J, Harris JA, Kennedy EM, Newell DG, Allen VM 2011 Longitudinal molecular epidemiological study of thermophilic campylobacters on one conventional broiler chicken farm. Appl Environ Microbiol 77:98–107. doi:10.1128/AEM.01388-10. 21037294 PMC3019741

[35] Sheppard SK, Colles F, Richardson J, Cody AJ, Elson R, Lawson A, Brick G, Meldrum R, Little CL, Owen RJ, Maiden MCJ, McCarthy ND 2010 Host association of Campylobacter genotypes transcends geographic variation. Appl Environ Microbiol 76:5269–5277. doi:10.1128/AEM.00124-10. 20525862 PMC2916502

[36] Oster RJ, Wijesinghe RU, Haack SK, Fogarty LR, Tucker TR, Riley SC 2014 Bacterial pathogen gene abundance and relation to recreational water quality at seven Great Lakes beaches. Environ Sci Technol 48:14148–14157. doi:10.1021/es5038657. 25423586

[37] Edwards DS, Milne LM, Morrow K, Sheridan P, Verlander NQ, Mulla R, Richardson JF, Pender A, Lilley M, Reacher M 2014 Campylobacteriosis outbreak associated with consumption of undercooked chicken liver pâté in the East of England, September 2011: identification of a dose-response risk. Epidemiol Infect 142:352–357. doi:10.1017/S0950268813001222. 23711104 PMC3891472

[38] Stucki U, Frey J, Nicolet J, Burnens AP 1995 Identification of Campylobacter jejuni on the basis of a species-specific gene that encodes a membrane protein. J Clin Microbiol 33:855–859. 7790451 10.1128/jcm.33.4.855-859.1995 PMC228055

[39] Johnson JG, Livny J, Dirita VJ 2014 High-throughput sequencing of Campylobacter jejuni insertion mutant libraries reveals *mapA* as a fitness factor for chicken colonization. J Bacteriol 196:1958–1967. doi:10.1128/JB.01395-13. 24633877 PMC4010991

[40] Shoaf-Sweeney KD, Larson CL, Tang X, Konkel ME 2008 Identification of Campylobacter jejuni proteins recognized by maternal antibodies of chickens. Appl Environ Microbiol 74:6867–6875. doi:10.1128/AEM.01097-08. 18805999 PMC2583476

[41] Richardson PT, Park SF 1995 Enterochelin acquisition in Campylobacter coli: characterization of components of a binding-protein-dependent transport system. Microbiology 141(Pt 12):3181–3191. doi:10.1099/13500872-141-12-3181. 8574410

[42] Cody AJ, McCarthy NM, Wimalarathna HL, Colles FM, Clark L, Bowler IC, Maiden MC, Dingle KE 2012 A longitudinal 6-year study of the molecular epidemiology of clinical Campylobacter isolates in Oxfordshire, United Kingdom. J Clin Microbiol 50:3193–3201. doi:10.1128/JCM.01086-12. 22814466 PMC3457434

[43] Sheppard SK, Dallas JF, MacRae M, McCarthy ND, Sproston EL, Gormley FJ, Strachan NJ, Ogden ID, Maiden MC, Forbes KJ 2009 Campylobacter genotypes from food animals, environmental sources and clinical disease in Scotland 2005/6. Int J Food Microbiol 134:96–103. doi:10.1016/j.ijfoodmicro.2009.02.010. 19269051 PMC3985063

[44] Dingle KE, Colles FM, Falush D, Maiden MC 2005 Sequence typing and comparison of population biology of Campylobacter coli and Campylobacter jejuni. J Clin Microbiol 43:340–347. doi:10.1128/JCM.43.1.340-347.2005. 15634992 PMC540151

[45] Dingle KE, Colles FM, Wareing DRA, Ure R, Fox AJ, Bolton FJ, Bootsma HJ, Willems RJL, Urwin R, Maiden MCJ 2001 Multilocus sequence typing system for Campylobacter jejuni. J Clin Microbiol 39:14–23. doi:10.1128/JCM.39.1.14-23.2001. 11136741 PMC87672

[46] Hunter PR, Gaston MA 1988 Numerical index of discriminatory ability of typing systems: an application of Simpson's index of diversity. J Clin Microbiol 26:2465–2466. 3069867 10.1128/jcm.26.11.2465-2466.1988 PMC266921

[47] Simpson EH 1949 Measurement of diversity. Nature 163:688. doi:10.1038/163688a0.

[48] Grundmann H, Hori S, Tanner G 2001 Determining confidence intervals when measuring genetic diversity and the discriminatory abilities of typing methods for microorganisms. J Clin Microbiol 39:4190–4192. doi:10.1128/JCM.39.11.4190-4192.2001. 11682558 PMC88515

[49] Katoh K, Standley DM 2013 MAFFT multiple sequence alignment software version 7: improvements in performance and usability. Mol Biol Evol 30:772–780. doi:10.1093/molbev/mst010. 23329690 PMC3603318

[50] Kumar S, Stecher G, Peterson D, Tamura K 2012 MEGA-CC: computing core of molecular evolutionary genetics analysis program for automated and iterative data analysis. Bioinformatics 28:2685–2686. doi:10.1093/bioinformatics/bts507. 22923298 PMC3467750

[51] Tamura K, Peterson D, Peterson N, Stecher G, Nei M, Kumar S 2011 MEGA5: molecular evolutionary genetics analysis using maximum likelihood, evolutionary distance, and maximum parsimony methods. Mol Biol Evol 28:2731–2739. doi:10.1093/molbev/msr121. 21546353 PMC3203626

[52] Saitou N, Nei M 1987 The neighbor-joining method: a new method for reconstructing phylogenetic trees. Mol Biol Evol 4:406–425. 3447015 10.1093/oxfordjournals.molbev.a040454

[53] Kimura M 1980 A simple method for estimating evolutionary rates of base substitutions through comparative studies of nucleotide sequences. J Mol Evol 16:111–120. doi:10.1007/BF01731581. 7463489

[54] Sheppard SK, Dallas JF, Wilson DJ, Strachan NJ, McCarthy ND, Colles FM, Rotariu O, Ogden ID, Forbes KJ, Maiden MCJ 2010 Evolution of an agriculture-associated disease causing Campylobacter coli clade: evidence from national surveillance data in Scotland. PLoS One 5:e15708. doi:10.1371/journal.pone.0015708. 21179537 PMC3002284

[55] Chen Y, Mukherjee S, Hoffmann M, Kotewicz ML, Young S, Abbott J, Luo Y, Davidson MK, Allard M, McDermott P, Zhao S 2013 Whole-genome sequencing of gentamicin-resistant Campylobacter coli isolated from U.S. retail meats reveals novel plasmid-mediated aminoglycoside resistance genes. Antimicrob Agents Chemother 57:5398–5405. doi:10.1128/AAC.00669-13. 23959310 PMC3811239

[56] Pearson BM, Rokney A, Crossman LC, Miller WG, Wain J, van Vliet AH 2013 Complete genome sequence of the Campylobacter coli clinical isolate 15-537360. Genome Announc 1:e01056–01013. doi:10.1128/genomeA.01056-13. 24336384 PMC3861437

[57] Pritchard JK, Stephens M, Donnelly P 2000 Inference of population structure using multilocus genotype data. Genetics 155:945–959. 10835412 10.1093/genetics/155.2.945 PMC1461096

[58] Falush D, Stephens M, Pritchard JK 2003 Inference of population structure using multilocus genotype data: linked loci and correlated allele frequencies. Genetics 164:1567–1587. 12930761 10.1093/genetics/164.4.1567 PMC1462648

[59] Librado P, Rozas J 2009 DnaSP v5: a software for comprehensive analysis of DNA polymorphism data. Bioinformatics 25:1451–1452. doi:10.1093/bioinformatics/btp187. 19346325

[60] Korber B 2000 HIV signature and sequence variation analysis, p 55–72. *In* Rodrigo AG, Learn GH (ed), Computational analysis of HIV molecular sequences. Kluwer Academic Publishers, Dordrecht, Netherlands.

[61] Miller CE, Williams PH, Ketley JM 2009 Pumping iron: mechanisms for iron uptake by Campylobacter. Microbiology 155:3157–3165. doi:10.1099/mic.0.032425-0. 19696110

[62] Palyada K, Threadgill D, Stintzi A 2004 Iron acquisition and regulation in Campylobacter jejuni. J Bacteriol 186:4714–4729. doi:10.1128/JB.186.14.4714-4729.2004. 15231804 PMC438614

[63] McCarthy ND, Colles FM, Dingle KE, Bagnall MC, Manning G, Maiden MC, Falush D 2007 Host-associated genetic import in Campylobacter jejuni. Emerg Infect Dis 13:267–272. doi:10.3201/eid1302.060620. 17479890 PMC2063414

[64] Rosef O, Gondrosen B, Kapperud G, Underdal B 1983 Isolation and characterization of Campylobacter jejuni and Campylobacter coli from domestic and wild mammals in Norway. Appl Environ Microbiol 46:855–859. 6639033 10.1128/aem.46.4.855-859.1983 PMC239479

[65] Waldenström J, Broman T, Carlsson I, Hasselquist D, Achterberg RP, Wagenaar JA, Olsen B 2002 Prevalence of Campylobacter jejuni, Campylobacter *lari*, and Campylobacter coli in different ecological guilds and taxa of migrating birds. Appl Environ Microbiol 68:5911–5917. doi:10.1128/AEM.68.12.5911-5917.2002. 12450810 PMC134389

[66] Parkhill J, Wren BW, Mungall K, Ketley JM, Churcher C, Basham D, Chillingworth T, Davies RM, Feltwell T, Holroyd S, Jagels K, Karlyshev AV, Moule S, Pallen MJ, Penn CW, Quail MA, Rajandream MA, Rutherford KM, van Vliet AH, Whitehead S, Barrell BG 2000 The genome sequence of the food-borne pathogen Campylobacter jejuni reveals hypervariable sequences. Nature 403:665–668. doi:10.1038/35001088. 10688204

[67] de Boer RF, Ott A, Guren P, van Zanten E, van Belkum A, Kooistra-Smid AM 2013 Detection of Campylobacter species and Arcobacter butzleri in stool samples by use of real-time multiplex PCR. J Clin Microbiol 51:253–259. doi:10.1128/JCM.01716-12. 23152553 PMC3536235

[68] de Boer RF, Ott A, Kesztyus B, Kooistra-Smid AM 2010 Improved detection of five major gastrointestinal pathogens by use of a molecular screening approach. J Clin Microbiol 48:4140–4146. doi:10.1128/JCM.01124-10. 20861334 PMC3020836

[69] Fukushima H, Katsube K, Tsunomori Y, Kishi R, Atsuta J, Akiba Y 2009 Comprehensive and rapid real-time PCR analysis of 21 foodborne outbreaks. Int J Microbiol 2009:917623. doi:10.1155/2009/917623. 20016673 PMC2775201

[70] McAuliffe GN, Anderson TP, Stevens M, Adams J, Coleman R, Mahagamasekera P, Young S, Henderson T, Hofmann M, Jennings LC, Murdoch DR 2013 Systematic application of multiplex PCR enhances the detection of bacteria, parasites, and viruses in stool samples. J Infect 67:122–129. doi:10.1016/j.jinf.2013.04.009. 23603249

