# Supplementary material for: Exploiting Bacterial Whole-Genome Sequencing Data for Evaluation of Diagnostic Assays: Campylobacter Species Identification as a Case Study
Source: J Clin Microbiol. 2016 Nov 23;54(12):2882–90. doi: 10.1128/JCM.01522-16 (PMC5121375; doi:10.1128/JCM.01522-16)
Supplement: Supplemental material [file supp_54_12_2882__index.html]

Supplemental material 

# Exploiting Bacterial Whole-Genome Sequencing Data for Evaluation of Diagnostic Assays: Campylobacter Species Identification as a Case Study

## Supplemental material

**Files in this Data Supplement:**

- Supplemental file 1 -

  Tables S1 (Details of isolates included in the *mapA* and *ceuE* evaluation project), S2 (Details of isolates used to confirm membership of *C. coli* clades 1, 2, and 3), and S3 (RT-PCR cycle thresholds for *mapA* and *ceuE* primer and probe combinations) and Fig. S1 (Frequency distribution of *mapA* and *ceuE* nucleotide and protein alleles in *C. jejuni* and *C. coli*) and S2 (Distribution of synonymous and nonsynonymous substitutions across *mapA* and *ceuE* in *C. jejuni* and *C. coli* as determined using SNAP)

  PDF, 909K
