# Supplementary material for: Exploiting Bacterial Whole-Genome Sequencing Data for Evaluation of Diagnostic Assays: Campylobacter Species Identification as a Case Study
Source: J Clin Microbiol. 2016 Nov 23;54(12):2882–90. doi: 10.1128/JCM.01522-16 (PMC5121375; doi:10.1128/JCM.01522-16)
Supplement: Supplemental material [file JCM.01522-16_zjm999095245so1.pdf]

SUPPLEMENTARY MATERIAL FOR

Exploiting bacterial whole-genome sequencing data for the evaluation of diagnostic assays: *Campylobacter* species identification as a case study.

This file contains:

Table S1
Table S2
Table S3
Fig. S1
Fig. S2

Table S1 Details of isolates included in the "*mapA*/*ceuE* Evaluation" project accessible at http://pubmlst.org/campylobacter/

PubMLST id	Isolate	ENA accession
10935	OXC6544	ERR108308
12880	OXC6545	ERR108309
12881	OXC6546	ERR108310
12882	OXC6547	ERR108311
12883	OXC6548	ERR108312
12884	OXC6549	ERR108313
12885	OXC6550	ERR108314
12886	OXC6551	ERR108315
12887	OXC6552	ERR108316
12888	OXC6553	ERR108317
12891	OXC6556	ERR108320
12892	OXC6557	ERR108321
12893	OXC6558	ERR108322
12894	OXC6559	ERR108323
12895	OXC6560	ERR108324
12896	OXC6561	ERR108325
12897	OXC6562	ERR108326
12898	OXC6563	ERR108327
12899	OXC6564	ERR108328
12900	OXC6565	ERR108329
12901	OXC6566	ERR108330
12902	OXC6567	ERR108331
12903	OXC6568	ERR108332
12904	OXC6569	ERR108333
12905	OXC6570	ERR108334
12906	OXC6571	ERR108335
12908	OXC6573	ERR108337
12909	OXC6574	ERR108338
12911	OXC6576	ERR108340
12912	OXC6577	ERR108341
12913	OXC6578	ERR108342
12916	OXC6579	ERR108343
12918	OXC6581	ERR108345
12919	OXC6582	ERR108346
12920	OXC6583	ERR108347
12921	OXC6584	ERR108348
12922	OXC6585	ERR108349
12923	OXC6586	ERR108350
12924	OXC6587	ERR108351

PubMLST id	Isolate	ENA accession
16048	OXC6250	ERR083867
16049	OXC6251	ERR083868
16050	OXC6252	ERR083869
16051	OXC6253	ERR083870
16052	OXC6254	ERR083871
16053	OXC6256	ERR083873
16054	OXC6257	ERR083874
16055	OXC6258	ERR083875
16056	OXC6259	ERR083876
16057	OXC6260	ERR083877
16058	OXC6261	ERR083878
16059	OXC6262	ERR083879
16060	OXC6263	ERR083880
16061	OXC6264	ERR083881
16062	OXC6265	ERR083882
16063	OXC6266	ERR083883
16064	OXC6267	ERR083884
16065	OXC6268	ERR083885
16066	OXC6269	ERR083886
16067	OXC6270	ERR083887
16068	OXC6271	ERR083888
16069	OXC6272	ERR083889
16071	OXC6274	ERR083891
16072	OXC6275	ERR083892
16073	OXC6276	ERR083893
16074	OXC6277	ERR083894
16075	OXC6278	ERR083895
16076	OXC6279	ERR083896
16078	OXC6281	ERR083897
16079	OXC6282	ERR083898
16080	OXC6283	ERR083899
16081	OXC6284	ERR083900
16082	OXC6285	ERR083901
16083	OXC6286	ERR083902
16084	OXC6287	ERR083903
16085	OXC6288	ERR083904
16086	OXC6289	ERR083905
16087	OXC6290	ERR083906
16088	OXC6291	ERR083907
16090	OXC6293	ERR083909
16091	OXC6294	ERR083910
16093	OXC6296	ERR083912

PubMLST id	Isolate	ENA accession
16094	OXC6297	ERR083913
16095	OXC6298	ERR083914
16097	OXC6300	ERR083916
16098	OXC6301	ERR083917
16100	OXC6303	ERR083919
16101	OXC6304	ERR083920
16102	OXC6305	ERR083921
16103	OXC6306	ERR083922
16104	OXC6307	ERR083923
16105	OXC6308	ERR083924
16106	OXC6309	ERR083925
16107	OXC6310	ERR083926
16108	OXC6311	ERR083927
16109	OXC6312	ERR083928
16110	OXC6313	ERR083929
16111	OXC6314	ERR083930
16112	OXC6315	ERR083931
16113	OXC6316	ERR083932
16114	OXC6317	ERR083933
16115	OXC6318	ERR083934
16116	OXC6319	ERR083935
16117	OXC6320	ERR083936
16118	OXC6321	ERR083937
16119	OXC6322	ERR083938
16120	OXC6323	ERR083939
16121	OXC6324	ERR083940
16122	OXC6325	ERR083941
16123	OXC6326	ERR083942
16124	OXC6327	ERR083943
16125	OXC6328	ERR083944
16126	OXC6329	ERR083945
16127	OXC6330	ERR083946
16128	OXC6331	ERR083947
16129	OXC6332	ERR083948
16130	OXC6333	ERR083949
16131	OXC6334	ERR083950
16132	OXC6335	ERR083951
16134	OXC6337	ERR083953
16135	OXC6338	ERR083954
16136	OXC6339	ERR083955
16137	OXC6340	ERR083956
16138	OXC6341	ERR083957

PubMLST id	Isolate	ENA accession
16139	OXC6342	ERR083958
16140	OXC6343	ERR083959
16141	OXC6344	ERR083960
16143	OXC6346	ERR083962
16145	OXC6348	ERR083964
16146	OXC6349	ERR083965
16147	OXC6350	ERR083966
16148	OXC6351	ERR083967
16149	OXC6352	ERR083968
16150	OXC6353	ERR083969
16151	OXC6354	ERR083970
16152	OXC6355	ERR083971
16153	OXC6356	ERR083972
16154	OXC6357	ERR083973
16155	OXC6358	ERR083974
16156	OXC6359	ERR083975
16157	OXC6360	ERR083976
16158	OXC6361	ERR083977
16159	OXC6362	ERR083978
16160	OXC6363	ERR083979
16161	OXC6364	ERR083980
16162	OXC6365	ERR083981
16163	OXC6366	ERR083982
16164	OXC6367	ERR083983
16165	OXC6368	ERR083984
16166	OXC6369	ERR083985
16167	OXC6370	ERR083986
16168	OXC6371	ERR083987
16169	OXC6372	ERR083988
16170	OXC6373	ERR083989
16171	OXC6374	ERR083990
16172	OXC6375	ERR083991
16173	OXC6376	ERR083992
16175	OXC6378	ERR083994
16177	OXC6380	ERR083996
16178	OXC6381	ERR083997
16179	OXC6382	ERR083998
16180	OXC6383	ERR083999
16181	OXC6384	ERR084000
16182	OXC6385	ERR084001
16183	OXC6386	ERR084002
16184	OXC6387	ERR084003

PubMLST id	Isolate	ENA accession
16186	OXC6389	ERR084005
16187	OXC6390	ERR084006
16188	OXC6391	ERR084007
16190	OXC6393	ERR084009
16191	OXC6394	ERR084010
16192	OXC6395	ERR084011
16193	OXC6396	ERR084012
16194	OXC6397	ERR084013
16195	OXC6400	ERR084014
16196	OXC6401	ERR084015
16197	OXC6402	ERR084016
16198	OXC6403	ERR084017
16200	OXC6405	ERR084019
16201	OXC6406	ERR084020
16202	OXC6407	ERR084021
16203	OXC6408	ERR084022
16204	OXC6409	ERR084023
16205	OXC6410	ERR084024
16206	OXC6411	ERR084025
16207	OXC6412	ERR084026
16208	OXC6413	ERR084027
16209	OXC6414	ERR084028
16210	OXC6415	ERR084029
16211	OXC6416	ERR084030
16212	OXC6417	ERR084031
16213	OXC6418	ERR084032
16214	OXC6419	ERR084033
16215	OXC6420	ERR084034
16216	OXC6421	ERR084035
16217	OXC6422	ERR084036
16218	OXC6423	ERR084037
16219	OXC6424	ERR084038
16220	OXC6425	ERR084039
16221	OXC6426	ERR084040
16222	OXC6427	ERR084041
16223	OXC6428	ERR084042
16224	OXC6429	ERR084043
16225	OXC6430	ERR084044
16226	OXC6431	ERR084045
16227	OXC6432	ERR084046
16228	OXC6433	ERR084047
16229	OXC6434	ERR084048

PubMLST id	Isolate	ENA accession
16231	OXC6436	ERR084050
16232	OXC6437	ERR084051
16233	OXC6438	ERR084052
16234	OXC6440	ERR084053
16235	OXC6441	ERR084054
16236	OXC6442	ERR084055
16239	OXC6446	ERR084058
16240	OXC6447	ERR084059
16241	OXC6448	ERR084060
16242	OXC6449	ERR084061
16243	OXC6450	ERR084062
16244	OXC6451	ERR084063
16245	OXC6453	ERR084064
16246	OXC6454	ERR084065
16247	OXC6455	ERR084066
16248	OXC6456	ERR084067
16249	OXC6457	ERR084068
16250	OXC6458	ERR084069
16251	OXC6459	ERR084070
16252	OXC6460	ERR084071
16253	OXC6461	ERR084072
16255	OXC6463	ERR084074
16256	OXC6464	ERR084075
16257	OXC6465	ERR084076
16258	OXC6466	ERR084077
16259	OXC6467	ERR084078
16260	OXC6468	ERR084079
16261	OXC6469	ERR084080
16262	OXC6470	ERR084081
16263	OXC6471	ERR084082
16265	OXC6473	ERR084084
16266	OXC6474	ERR084085
16267	OXC6475	ERR084086
16268	OXC6476	ERR084087
16269	OXC6477	ERR084088
16270	OXC6478	ERR084089
16271	OXC6479	ERR084090
16272	OXC6480	ERR084091
16273	OXC6481	ERR084092
16274	OXC6482	ERR084093
16275	OXC6483	ERR084094
16276	OXC6484	ERR084095

PubMLST id	Isolate	ENA accession
16278	OXC6486	ERR084097
16279	OXC6487	ERR084098
16280	OXC6488	ERR084099
16281	OXC6489	ERR084100
16282	OXC6490	ERR084101
16283	OXC6491	ERR084102
16284	OXC6492	ERR084103
16285	OXC6493	ERR084104
16286	OXC6494	ERR084105
16287	OXC6495	ERR084106
16288	OXC6496	ERR084107
16289	OXC6497	ERR084108
16290	OXC6498	ERR084109
16291	OXC6499	ERR084110
16292	OXC6500	ERR084111
16293	OXC6501	ERR084112
16294	OXC6502	ERR084113
16295	OXC6503	ERR084114
16296	OXC6504	ERR084115
16297	OXC6505	ERR084116
16298	OXC6506	ERR084117
16299	OXC6507	ERR084118
16300	OXC6508	ERR084119
16301	OXC6509	ERR084120
16302	OXC6510	ERR084121
16303	OXC6511	ERR084122
16304	OXC6512	ERR084123
16305	OXC6513	ERR084124
16306	OXC6514	ERR084125
16307	OXC6515	ERR084126
16308	OXC6516	ERR084127
16309	OXC6517	ERR084128
16310	OXC6518	ERR084129
16311	OXC6519	ERR084130
16312	OXC6520	ERR084131
16313	OXC6521	ERR084132
16314	OXC6522	ERR084133
16315	OXC6523	ERR084134
16316	OXC6524	ERR084135
16317	OXC6525	ERR084136
16318	OXC6526	ERR084137
16319	OXC6527	ERR084138

PubMLST id	Isolate	ENA accession
16320	OXC6528	ERR084139
16321	OXC6529	ERR084140
16322	OXC6530	ERR084141
16323	OXC6531	ERR084142
16324	OXC6532	ERR084143
16325	OXC6533	ERR084144
16326	OXC6534	ERR084145
16327	OXC6535	ERR084146
16328	OXC6536	ERR084147
16329	OXC6537	ERR084148
16330	OXC6538	ERR084149
16331	OXC6539	ERR084150
16332	OXC6540	ERR084151
16333	OXC6541	ERR084152
16334	OXC6542	ERR084153
16335	OXC6543	ERR084154
16336	OXC6255	ERR083872
16341	OXC6590	ERR108354
16342	OXC6591	ERR108355
16343	OXC6592	ERR108356
16345	OXC6594	ERR108358
16346	OXC6595	ERR108359
16347	OXC6596	ERR108360
16348	OXC6597	ERR108361
16349	OXC6598	ERR108362
16350	OXC6599	ERR108363
16351	OXC6600	ERR108364
16352	OXC6601	ERR108365
16353	OXC6602	ERR108366
16354	OXC6603	ERR108367
16355	OXC6604	ERR108368
16357	OXC6607	ERR108370
16358	OXC6608	ERR108371
16359	OXC6609	ERR108372
16361	OXC6611	ERR108374
16362	OXC6612	ERR108375
16363	OXC6613	ERR108376
16364	OXC6614	ERR108377
16365	OXC6615	ERR108378
16366	OXC6616	ERR108379
16367	OXC6618	ERR108380
16368	OXC6619	ERR108381

PubMLST id	Isolate	ENA accession
16369	OXC6620	ERR108382
16370	OXC6621	ERR108383
16371	OXC6622	ERR108384
16372	OXC6623	ERR108385
16373	OXC6624	ERR108386
16374	OXC6625	ERR108387
16375	OXC6626	ERR108388
16376	OXC6627	ERR108389
16377	OXC6628	ERR108390
16378	OXC6629	ERR108391
16379	OXC6630	ERR108392
16380	OXC6631	ERR108393
16381	OXC6632	ERR108394
16382	OXC6633	ERR108395
16383	OXC6634	ERR108396
16384	OXC6635	ERR108397
16385	OXC6636	ERR108398
16386	OXC6637	ERR108399
16387	OXC6638	ERR108400
16388	OXC6639	ERR108401
16389	OXC6640	ERR108402
16390	OXC6641	ERR108403
18199	OXC6642	ERR128727
18200	OXC6643	ERR128728
18201	OXC6644	ERR128729
18202	OXC6645	ERR128730
18203	OXC6646	ERR128731
18204	OXC6647	ERR128732
18205	OXC6648	ERR128733
18206	OXC6649	ERR128734
18207	OXC6650	ERR128735
18208	OXC6651	ERR128736
18209	OXC6652	ERR128737
18210	OXC6653	ERR128738
18211	OXC6654	ERR128739
18212	OXC6655	ERR128740
18213	OXC6656	ERR128741
18214	OXC6657	ERR128742
18216	OXC6659	ERR128744
18217	OXC6660	ERR128745
18218	OXC6661	ERR128746
18219	OXC6662	ERR128747

PubMLST id	Isolate	ENA accession
18220	OXC6663	ERR128748
18221	OXC6664	ERR128749
18222	OXC6665	ERR128750
18223	OXC6666	ERR128751
18224	OXC6667	ERR128752
18225	OXC6668	ERR128753
18226	OXC6669	ERR128754
18228	OXC6671	ERR128756
18229	OXC6672	ERR128757
18230	OXC6673	ERR128758
18231	OXC6674	ERR128759
18232	OXC6675	ERR128760
18233	OXC6676	ERR128761
18234	OXC6677	ERR128762
18235	OXC6678	ERR128763
18236	OXC6679	ERR128764
18237	OXC6680	ERR128765
18238	OXC6681	ERR128766
18239	OXC6682	ERR128767
18240	OXC6683	ERR128768
18241	OXC6684	ERR128769
18242	OXC6685	ERR128770
18245	OXC6688	ERR128773
18246	OXC6689	ERR128774
18247	OXC6690	ERR128775
18248	OXC6691	ERR128776
18250	OXC6693	ERR128778
18251	OXC6694	ERR128779
18252	OXC6695	ERR128780
18253	OXC6696	ERR128781
18254	OXC6697	ERR128782
18255	OXC6698	ERR128783
18256	OXC6699	ERR128784
18257	OXC6700	ERR128785
18258	OXC6701	ERR128786
18259	OXC6702	ERR128787
18260	OXC6703	ERR128788
18262	OXC6705	ERR128790
18263	OXC6706	ERR128791
18264	OXC6707	ERR128792
18265	OXC6708	ERR128793
18266	OXC6709	ERR128794

PubMLST id	Isolate	ENA accession
18267	OXC6710	ERR128795
18268	OXC6711	ERR128796
18270	OXC6713	ERR128798
18271	OXC6714	ERR128799
18272	OXC6715	ERR128800
18273	OXC6716	ERR128801
18275	OXC6718	ERR128803
18276	OXC6719	ERR128804
18278	OXC6721	ERR128806
18279	OXC6722	ERR128807
18280	OXC6723	ERR128808
18281	OXC6724	ERR128809
18282	OXC6725	ERR128810
18283	OXC6726	ERR128811
18284	OXC6727	ERR128812
18285	OXC6728	ERR128813
18286	OXC6729	ERR128814
18287	OXC6730	ERR128815
18288	OXC6731	ERR128816
18289	OXC6732	ERR128817
18290	OXC6733	ERR128818
18291	OXC6734	ERR128819
18292	OXC6735	ERR128820
18293	OXC6736	ERR128821
18294	OXC6737	ERR128822
18295	OXC6738	ERR136999
18296	OXC6739	ERR137000
18297	OXC6740	ERR137001
18298	OXC6741	ERR137002
18299	OXC6742	ERR137003
18300	OXC6743	ERR137004
18301	OXC6744	ERR137005
18302	OXC6745	ERR137006
18303	OXC6746	ERR137007
18304	OXC6747	ERR137008
18305	OXC6748	ERR137009
18306	OXC6749	ERR137010
18307	OXC6750	ERR137011
18308	OXC6751	ERR137012
18309	OXC6752	ERR137013
18310	OXC6753	ERR137014
18311	OXC6754	ERR137015

PubMLST id	Isolate	ENA accession
18312	OXC6755	ERR137016
18313	OXC6756	ERR137017
18314	OXC6757	ERR137018
18316	OXC6759	ERR137020
18317	OXC6760	ERR137021
18318	OXC6761	ERR137022
18319	OXC6762	ERR137023
18320	OXC6763	ERR137024
18321	OXC6764	ERR137025
18322	OXC6765	ERR137026
18323	OXC6766	ERR137027
18324	OXC6767	ERR137028
18325	OXC6768	ERR137029
18326	OXC6769	ERR137030
18327	OXC6770	ERR137031
18328	OXC6771	ERR137032
18329	OXC6772	ERR137033
18330	OXC6773	ERR137034
18331	OXC6774	ERR137035
18333	OXC6776	ERR137037
18334	OXC6777	ERR137038
18335	OXC6778	ERR137039
18336	OXC6779	ERR137040
18337	OXC6780	ERR137041
18338	OXC6781	ERR137042
18340	OXC6783	ERR137044
18342	OXC6785	ERR137046
18343	OXC6786	ERR137047
18344	OXC6787	ERR137048
18345	OXC6788	ERR137049
18346	OXC6789	ERR137050
18347	OXC6790	ERR137051
18348	OXC6791	ERR137052
18350	OXC6793	ERR137054
18352	OXC6795	ERR137056
18353	OXC6796	ERR137057
18354	OXC6797	ERR137058
18355	OXC6798	ERR137059
18356	OXC6799	ERR137060
18357	OXC6800	ERR137061
18358	OXC6801	ERR137062
18359	OXC6802	ERR137063

PubMLST id	Isolate	ENA accession
18360	OXC6803	ERR137064
18361	OXC6804	ERR137065
18362	OXC6805	ERR137066
18363	OXC6806	ERR137067
18364	OXC6807	ERR137068
18365	OXC6808	ERR137069
18366	OXC6809	ERR137070
18367	OXC6810	ERR137071
18368	OXC6811	ERR137072
18369	OXC6812	ERR137073
18370	OXC6813	ERR137074
18371	OXC6814	ERR137075
18372	OXC6815	ERR137076
18373	OXC6816	ERR137077
18374	OXC6817	ERR137078
18375	OXC6818	ERR137079
18376	OXC6819	ERR137080
18377	OXC6820	ERR137081
18378	OXC6821	ERR137082
18380	OXC6823	ERR137084
18381	OXC6824	ERR137085
18382	OXC6825	ERR137086
18383	OXC6826	ERR137087
18384	OXC6827	ERR137088
18385	OXC6828	ERR137089
18386	OXC6830	ERR137090
18387	OXC6831	ERR137091
18388	OXC6832	ERR137092
18389	OXC6833	ERR137093
18390	OXC6834	ERR137094
21106	OXC6932	ERR189774
21107	OXC6933	ERR189775
21108	OXC6934	ERR189776
21109	OXC6935	ERR189777
21110	OXC6936	ERR189778
21111	OXC6937	ERR189779
21112	OXC6938	ERR189780
21113	OXC6939	ERR189781
21114	OXC6940	ERR189782
21115	OXC6941	ERR189783
21116	OXC6942	ERR189784
21117	OXC6943	ERR189785

PubMLST id	Isolate	ENA accession
21118	OXC6944	ERR189786
21119	OXC6945	ERR189787
21120	OXC6946	ERR189788
21121	OXC6947	ERR189789
21122	OXC6948	ERR189790
21123	OXC6949	ERR189791
21124	OXC6950	ERR189792
21125	OXC6951	ERR189793
21126	OXC6952	ERR189794
21127	OXC6953	ERR189795
21128	OXC6954	ERR189796
21129	OXC6955	ERR189797
21130	OXC6956	ERR189798
21131	OXC6957	ERR189799
21132	OXC6958	ERR189800
21133	OXC6959	ERR189801
21134	OXC6960	ERR189802
21135	OXC6961	ERR189803
21136	OXC6962	ERR189804
21137	OXC6963	ERR189805
21138	OXC6964	ERR189806
21139	OXC6965	ERR189807
21140	OXC6966	ERR189808
21141	OXC6967	ERR189809
21142	OXC6968	ERR189810
21143	OXC6969	ERR189811
21146	OXC6973	ERR189814
21147	OXC6974	ERR189815
21149	OXC6976	ERR189817
21150	OXC6977	ERR189818
21151	OXC6978	ERR189819
21152	OXC6979	ERR189820
21153	OXC6980	ERR189821
21154	OXC6981	ERR189822
21155	OXC6982	ERR189823
21156	OXC6983	ERR189824
21157	OXC6984	ERR189825
21158	OXC6985	ERR189826
21160	OXC6987	ERR189828
21161	OXC6988	ERR189829
21162	OXC6989	ERR189830
21163	OXC6991	ERR189831

PubMLST id	Isolate	ENA accession
21164	OXC6992	ERR189861
21165	OXC6994	ERR189862
21167	OXC6996	ERR189864
21168	OXC6997	ERR189865
21169	OXC6998	ERR189866
21170	OXC6999	ERR189867
21171	OXC7000	ERR189868
21172	OXC7001	ERR189869
21183	OXC7101	ERR193150
21184	OXC7102	ERR193151
21185	OXC7103	ERR193152
21186	OXC7104	ERR193153
21187	OXC7105	ERR193154
21188	OXC7106	ERR193155
21189	OXC7107	ERR193156
21190	OXC7108	ERR193157
21191	OXC7109	ERR193158
21192	OXC7110	ERR193159
21193	OXC7111	ERR193160
21194	OXC7112	ERR193161
21195	OXC7113	ERR193162
21196	OXC7114	ERR193163
21197	OXC7115	ERR193164
21198	OXC7116	ERR193165
21199	OXC7117	ERR193166
21201	OXC7119	ERR193168
21202	OXC7120	ERR193169
21203	OXC7121	ERR193170
21204	OXC7122	ERR193171
21205	OXC7123	ERR193172
21206	OXC7124	ERR193173
21207	OXC7125	ERR193174
21208	OXC7127	ERR193175
21209	OXC7128	ERR193176
21210	OXC7129	ERR193177
21211	OXC7130	ERR193178
21212	OXC7131	ERR193179
21213	OXC7132	ERR193180
21214	OXC7133	ERR193181
21215	OXC7134	ERR193182
21216	OXC7135	ERR193183
21217	OXC7136	ERR193184

PubMLST id	Isolate	ENA accession
21219	OXC7138	ERR193186
21220	OXC7139	ERR193187
21221	OXC7140	ERR193188
21222	OXC7141	ERR193189
21223	OXC7142	ERR193190
21224	OXC7143	ERR193191
21225	OXC7144	ERR193192
21226	OXC7145	ERR193193
21227	OXC7146	ERR193194
21228	OXC7147	ERR193195
21229	OXC7148	ERR193196
21230	OXC7149	ERR193197
21231	OXC7150	ERR193198
21232	OXC7151	ERR193199
21234	OXC7153	ERR193201
21235	OXC7154	ERR193202
21236	OXC7155	ERR193203
21237	OXC7156	ERR193204
21238	OXC7157	ERR193205
21239	OXC7158	ERR193206
21240	OXC7159	ERR193207
21241	OXC7160	ERR193208
21242	OXC7161	ERR193209
21243	OXC7162	ERR193210
21244	OXC7163	ERR193211
21245	OXC7164	ERR193212
21246	OXC7165	ERR193213
21247	OXC7166	ERR193214
21248	OXC7167	ERR193215
21249	OXC7168	ERR193216
21250	OXC7169	ERR193217
21507	OXC6593	ERR108357
21554	OXC6835	ERR356037
21555	OXC6836	ERR356038
21556	OXC6837	ERR356039
21557	OXC6838	ERR356040
21558	OXC6839	ERR356041
21559	OXC6840	ERR356042
21560	OXC6841	ERR356043
21561	OXC6842	ERR356044
21562	OXC6843	ERR356045
21563	OXC6844	ERR356046

PubMLST id	Isolate	ENA accession
21564	OXC6845	ERR356047
21565	OXC6846	ERR356048
21566	OXC6847	ERR356049
21567	OXC6848	ERR356050
21568	OXC6849	ERR356051
21569	OXC6850	ERR356052
21570	OXC6851	ERR356053
21571	OXC6852	ERR356054
21572	OXC6853	ERR356055
21573	OXC6854	ERR356056
21574	OXC6855	ERR356057
21575	OXC6856	ERR356058
21576	OXC6857	ERR356059
21577	OXC6858	ERR356060
21578	OXC6859	ERR356061
21579	OXC6860	ERR356062
21580	OXC6861	ERR356063
21581	OXC6862	ERR356064
21582	OXC6863	ERR356065
21583	OXC6864	ERR356066
21584	OXC6865	ERR356067
21585	OXC6866	ERR356068
21586	OXC6867	ERR356069
21587	OXC6868	ERR356070
21588	OXC6869	ERR356071
21589	OXC6870	ERR356072
21590	OXC6871	ERR356073
21591	OXC6872	ERR356074
21592	OXC6873	ERR356075
21593	OXC6874	ERR356076
21594	OXC6875	ERR356077
21595	OXC6876	ERR356078
21596	OXC6877	ERR356079
21597	OXC6878	ERR356080
21598	OXC6879	ERR356081
21599	OXC6880	ERR356082
21600	OXC6881	ERR356083
22078	OXC6882	ERR356084
22079	OXC6883	ERR356085
22080	OXC6884	ERR356086
22081	OXC6885	ERR356087
22082	OXC6886	ERR356088

PubMLST id	Isolate	ENA accession
22083	OXC6887	ERR356089
22084	OXC6888	ERR356090
22085	OXC6890	ERR356091
22086	OXC6891	ERR356092
22087	OXC6892	ERR356093
22088	OXC6893	ERR356094
22089	OXC6894	ERR356095
22090	OXC6895	ERR356096
22092	OXC6897	ERR356098
22093	OXC6898	ERR356099
22094	OXC6899	ERR356100
22095	OXC6900	ERR356101
22096	OXC6901	ERR356102
22097	OXC6902	ERR356103
22098	OXC6903	ERR356104
22099	OXC6904	ERR356105
22100	OXC6905	ERR356106
22101	OXC6906	ERR356107
22102	OXC6907	ERR356108
22103	OXC6908	ERR356109
22104	OXC6909	ERR356110
22105	OXC6910	ERR356111
22107	OXC6912	ERR356113
22108	OXC6913	ERR356114
22109	OXC6914	ERR356115
22110	OXC6915	ERR356116
22111	OXC6916	ERR356117
22112	OXC6917	ERR356118
22113	OXC6918	ERR356119
22114	OXC6919	ERR356120
22115	OXC6920	ERR356121
22116	OXC6921	ERR356122
22117	OXC6922	ERR356123
22118	OXC6923	ERR356124
22119	OXC6924	ERR356125
22120	OXC6925	ERR356126
22121	OXC6926	ERR356127
22122	OXC6927	ERR356128
22123	OXC6928	ERR356129
22124	OXC6929	ERR356130
22125	OXC6930	ERR356131
22189	OXC7002	ERR221286

PubMLST id	Isolate	ENA accession
22190	OXC7003	ERR221287
22191	OXC7004	ERR221288
22192	OXC7005	ERR221289
22193	OXC7006	ERR221290
22194	OXC7007	ERR221291
22195	OXC7008	ERR221292
22196	OXC7009	ERR221293
22197	OXC7010	ERR221294
22198	OXC7011	ERR221295
22199	OXC7012	ERR221296
22200	OXC7013	ERR221297
22201	OXC7014	ERR221298
22202	OXC7015	ERR221299
22203	OXC7016	ERR221300
22204	OXC7017	ERR221301
22205	OXC7018	ERR221302
22206	OXC7019	ERR221303
22207	OXC7020	ERR221304
22209	OXC7022	ERR221306
22211	OXC7024	ERR221308
22212	OXC7025	ERR221309
22213	OXC7026	ERR221310
22214	OXC7027	ERR221311
22215	OXC7029	ERR221312
22216	OXC7030	ERR221313
22217	OXC7031	ERR221314
22218	OXC7032	ERR221315
22219	OXC7033	ERR221316
22220	OXC7034	ERR221317
22221	OXC7035	ERR221318
22222	OXC7036	ERR221319
22223	OXC7037	ERR221320
22224	OXC7038	ERR221321
22225	OXC7039	ERR221322
22226	OXC7041	ERR221323
22228	OXC7043	ERR221325
22229	OXC7044	ERR221326
22230	OXC7045	ERR221327
22231	OXC7046	ERR221328
22232	OXC7047	ERR221329
22233	OXC7048	ERR221330
22234	OXC7049	ERR221331

PubMLST id	Isolate	ENA accession
22235	OXC7050	ERR221332
22237	OXC7052	ERR221334
22238	OXC7053	ERR221335
22239	OXC7054	ERR221336
22241	OXC7056	ERR221338
22242	OXC7057	ERR221339
22243	OXC7058	ERR221340
22244	OXC7060	ERR221341
22245	OXC7061	ERR221342
22246	OXC7062	ERR221343
22247	OXC7063	ERR221344
22248	OXC7064	ERR221345
22249	OXC7065	ERR221346
22250	OXC7066	ERR221347
22251	OXC7067	ERR221348
22252	OXC7068	ERR221349
22253	OXC7069	ERR221350
22254	OXC7070	ERR221351
22255	OXC7071	ERR221352
22256	OXC7072	ERR221353
22257	OXC7073	ERR221354
22258	OXC7074	ERR221355
22259	OXC7075	ERR221356
22260	OXC7076	ERR221357
22261	OXC7077	ERR221358
22262	OXC7078	ERR221359
22263	OXC7079	ERR221360
22264	OXC7080	ERR221361
22265	OXC7081	ERR221362
22267	OXC7083	ERR221364
22268	OXC7084	ERR221365
22269	OXC7085	ERR221366
22270	OXC7086	ERR221367
22271	OXC7087	ERR221368
22272	OXC7090	ERR221369
22273	OXC7091	ERR221370
22274	OXC7092	ERR221371
22275	OXC7093	ERR221372
22277	OXC7095	ERR221374
22278	OXC7096	ERR221375
22279	OXC7097	ERR221376
22280	OXC7098	ERR221377

PubMLST id	Isolate	ENA accession
22281	OXC7099	ERR221378
22282	OXC7100	ERR221379
22283	OXC7241	ERR221380
22284	OXC7242	ERR221381
22285	OXC7243	ERR221382
22287	OXC7245	ERR221384
22288	OXC7246	ERR221385
22289	OXC7247	ERR221386
22290	OXC7248	ERR221387
22291	OXC7249	ERR221388
22292	OXC7250	ERR221389
22293	OXC7251	ERR221390
22294	OXC7252	ERR221391
22295	OXC7253	ERR221392
22296	OXC7255	ERR221393
22297	OXC7256	ERR221394
22298	OXC7257	ERR221395
22299	OXC7258	ERR221396
22300	OXC7259	ERR221397
22301	OXC7260	ERR221398
22302	OXC7261	ERR221399
22303	OXC7262	ERR221400
22304	OXC7263	ERR221401
22305	OXC7264	ERR221402
22306	OXC7265	ERR221403
22307	OXC7266	ERR221404
22308	OXC7267	ERR221405
22309	OXC7268	ERR221406
22310	OXC7269	ERR221407
22311	OXC7270	ERR221408
22312	OXC7271	ERR221409
22313	OXC7272	ERR221410
22314	OXC7273	ERR221411
22315	OXC7274	ERR221412
22316	OXC7275	ERR221413
22317	OXC7276	ERR221414
22318	OXC7277	ERR221415
22319	OXC7278	ERR221416
22320	OXC7279	ERR221417
22321	OXC7280	ERR221418
22322	OXC7281	ERR221419
22323	OXC7282	ERR221420

PubMLST id	Isolate	ENA accession
22324	OXC7283	ERR221421
22325	OXC7284	ERR221422
22326	OXC7285	ERR221423
22327	OXC7286	ERR221424
22328	OXC7287	ERR221425
22329	OXC7288	ERR221426
22330	OXC7289	ERR221427
22331	OXC7290	ERR221428
22332	OXC7291	ERR221429
22333	OXC7292	ERR221430
22334	OXC7293	ERR221431
22335	OXC7294	ERR221432
22336	OXC7295	ERR221433
22337	OXC7296	ERR221434
22338	OXC7297	ERR221435
22339	OXC7298	ERR221436
22340	OXC7299	ERR221437
22341	OXC7300	ERR221438
22342	OXC7301	ERR221439
22343	OXC7302	ERR221440
22344	OXC7303	ERR221441
22345	OXC7304	ERR221442
22346	OXC7305	ERR221443
22347	OXC7306	ERR221444
22348	OXC7307	ERR221445
22349	OXC7308	ERR221446
22350	OXC7309	ERR221447
22351	OXC7310	ERR221448
22352	OXC7311	ERR221449
22353	OXC7312	ERR221450
22354	OXC7313	ERR221451
22355	OXC7314	ERR221452
22356	OXC7315	ERR221453
22357	OXC7316	ERR221454
22358	OXC7319	ERR221455
22359	OXC7320	ERR221456
22360	OXC7321	ERR221457
22361	OXC7322	ERR221458
22362	OXC7323	ERR221459
22363	OXC7324	ERR221460
22364	OXC7325	ERR221461
22365	OXC7326	ERR221462

PubMLST id	Isolate	ENA accession
22366	OXC7328	ERR221463
22367	OXC7329	ERR221464
22368	OXC7330	ERR221465
22369	OXC7331	ERR221466
22370	OXC7332	ERR221467
22371	OXC7333	ERR221468
22372	OXC7334	ERR221469
22373	OXC7335	ERR221470
22374	OXC7336	ERR221471
22375	OXC7338	ERR221472
22376	OXC7339	ERR221473
22377	OXC7340	ERR221474
22378	OXC7341	ERR221475
22660	OXC7089	ERR222683
22661	OXC7170	ERR222684
22662	OXC7171	ERR222685
22664	OXC7173	ERR222687
22665	OXC7174	ERR222688
22666	OXC7175	ERR222689
22667	OXC7176	ERR222690
22668	OXC7177	ERR222691
22669	OXC7178	ERR222692
22670	OXC7179	ERR222693
22671	OXC7180	ERR222694
22672	OXC7181	ERR222695
22673	OXC7182	ERR222696
22674	OXC7183	ERR222697
22675	OXC7184	ERR222698
22676	OXC7185	ERR222699
22677	OXC7186	ERR222700
22678	OXC7187	ERR222701
22679	OXC7188	ERR222702
22680	OXC7189	ERR222703
22681	OXC7190	ERR222704
22682	OXC7191	ERR222705
22683	OXC7192	ERR222706
22684	OXC7193	ERR222707
22685	OXC7194	ERR222708
22686	OXC7195	ERR222709
22687	OXC7196	ERR222710
22688	OXC7197	ERR222711
22689	OXC7198	ERR222712

PubMLST id	Isolate	ENA accession
22690	OXC7199	ERR222713
22691	OXC7200	ERR222714
22692	OXC7201	ERR222715
22693	OXC7202	ERR222716
22694	OXC7203	ERR222717
22695	OXC7204	ERR222718
22696	OXC7205	ERR222719
22697	OXC7206	ERR222720
22698	OXC7207	ERR222721
22699	OXC7208	ERR222722
22700	OXC7209	ERR222723
22701	OXC7210	ERR222724
22702	OXC7211	ERR222725
22703	OXC7212	ERR222726
22704	OXC7213	ERR222727
22705	OXC7214	ERR222728
22706	OXC7215	ERR222729
22707	OXC7216	ERR222730
22708	OXC7217	ERR222731
22709	OXC7218	ERR222732
22710	OXC7219	ERR222733
22711	OXC7220	ERR222734
22712	OXC7221	ERR222735
22713	OXC7222	ERR222736
22714	OXC7223	ERR222737
22715	OXC7224	ERR222738
22716	OXC7225	ERR222739
22717	OXC7226	ERR222740
22718	OXC7227	ERR222741
22719	OXC7228	ERR222742
22720	OXC7230	ERR222743
22721	OXC7231	ERR222744
22722	OXC7232	ERR222745
22723	OXC7233	ERR222746
22724	OXC7234	ERR222747
22725	OXC7235	ERR222748
22726	OXC7236	ERR222749
22727	OXC7237	ERR222750
22728	OXC7238	ERR222751
22729	OXC7239	ERR222752
22730	OXC7240	ERR222753
22905	OXC6605	ERR108369

PubMLST id	Isolate	ENA accession
23883	OXC7342	ERR278228
23884	OXC7344	ERR278229
23885	OXC7345	ERR278230
23886	OXC7346	ERR278231
23887	OXC7347	ERR278232
23888	OXC7348	ERR278233
23889	OXC7349	ERR278234
23890	OXC7350	ERR278235
23891	OXC7351	ERR278236
23892	OXC7353	ERR278237
23893	OXC7354	ERR278238
23894	OXC7355	ERR278239
23895	OXC7356	ERR278240
23896	OXC7357	ERR278241
23897	OXC7358	ERR278242
23898	OXC7359	ERR278243
23899	OXC7360	ERR278244
23900	OXC7361	ERR278245
23901	OXC7362	ERR278246
23902	OXC7363	ERR278247
23903	OXC7364	ERR278248
23904	OXC7365	ERR278249
23905	OXC7366	ERR278250
23906	OXC7367	ERR278251
23907	OXC7369	ERR278252
23908	OXC7370	ERR278253
23909	OXC7371	ERR278254
23910	OXC7372	ERR278255
23911	OXC7373	ERR278256
23912	OXC7374	ERR278257
23913	OXC7375	ERR278258
23914	OXC7376	ERR278259
23915	OXC7377	ERR278260
23916	OXC7378	ERR278261
23917	OXC7379	ERR278262
23918	OXC7380	ERR278263
23919	OXC7381	ERR278264
23920	OXC7382	ERR278265
23921	OXC7383	ERR278266
23922	OXC7384	ERR278267
23923	OXC7385	ERR278268
23924	OXC7386	ERR278269

PubMLST id	Isolate	ENA accession
23925	OXC7387	ERR278270
23926	OXC7388	ERR278271
23927	OXC7389	ERR278272
23928	OXC7390	ERR278273
23929	OXC7391	ERR278274
23930	OXC7392	ERR278275
23932	OXC7394	ERR278277
23933	OXC7395	ERR278278
23934	OXC7397	ERR278279
23935	OXC7398	ERR278280
23936	OXC7400	ERR278281
23937	OXC7401	ERR278282
23940	OXC7404	ERR278285
23941	OXC7405	ERR278286
23942	OXC7407	ERR278287
23943	OXC7408	ERR278288
23944	OXC7409	ERR278289
23945	OXC7410	ERR278322
23946	OXC7411	ERR278323
23947	OXC7412	ERR278324
23948	OXC7414	ERR278325
23949	OXC7415	ERR278326
23950	OXC7416	ERR278327
23951	OXC7417	ERR278328
23952	OXC7418	ERR278329
23954	OXC7420	ERR278331
23955	OXC7421	ERR278332
23956	OXC7422	ERR278333
23957	OXC7423	ERR278334
23958	OXC7424	ERR278335
23959	OXC7425	ERR278336
23961	OXC7428	ERR278338
23962	OXC7429	ERR278339
23963	OXC7430	ERR278340
23964	OXC7431	ERR278341
23965	OXC7432	ERR278342
23966	OXC7433	ERR278343
23967	OXC7436	ERR278344
23968	OXC7437	ERR278345
23969	OXC7438	ERR278346
23970	OXC7439	ERR278347
23971	OXC7440	ERR278348

PubMLST id	Isolate	ENA accession
23972	OXC7441	ERR278349
23973	OXC7442	ERR278350
23974	OXC7443	ERR278351
23975	OXC7444	ERR278352
23976	OXC7446	ERR278353
23977	OXC7447	ERR278354
23978	OXC7448	ERR278355
23979	OXC7449	ERR278356
23980	OXC7450	ERR278357
23981	OXC7451	ERR278358
23982	OXC7452	ERR278359
23983	OXC7453	ERR278360
23984	OXC7454	ERR278361
23985	OXC7455	ERR278362
23986	OXC7456	ERR278363
23987	OXC7457	ERR278364
23988	OXC7458	ERR278365
23989	OXC7459	ERR278366
23990	OXC7461	ERR278367
23991	OXC7462	ERR278368
23992	OXC7463	ERR278369
23993	OXC7464	ERR278370
23994	OXC7465	ERR278371
23995	OXC7466	ERR278372
23996	OXC7467	ERR278373
23997	OXC7468	ERR278374
23998	OXC7469	ERR278375
23999	OXC7470	ERR278376
24000	OXC7471	ERR278377
24001	OXC7472	ERR278378
24002	OXC7473	ERR278379
24003	OXC7474	ERR278380
24004	OXC7475	ERR278381
24005	OXC7476	ERR278382
24006	OXC7477	ERR278383
24007	OXC7478	ERR278384
24008	OXC7479	ERR278385
24009	OXC7480	ERR278386
24010	OXC7481	ERR278387
24011	OXC7482	ERR278388
24012	OXC7484	ERR278389
24013	OXC7485	ERR278390

PubMLST id	Isolate	ENA accession
24014	OXC7486	ERR278391
24015	OXC7487	ERR278392
24016	OXC7488	ERR278393
24017	OXC7489	ERR278394
24018	OXC7490	ERR278395
24019	OXC7491	ERR278396
24021	OXC7493	ERR278398
24022	OXC7494	ERR278399
24023	OXC7495	ERR278400
24024	OXC7496	ERR278401
24025	OXC7498	ERR278402
24026	OXC7499	ERR278403
24027	OXC7500	ERR278404
24028	OXC7502	ERR278405
24029	OXC7503	ERR278406
24030	OXC7504	ERR278407
24031	OXC7505	ERR278408
24032	OXC7506	ERR278409
24033	OXC7507	ERR278410
24034	OXC7508	ERR278411
24035	OXC7509	ERR278412
24036	OXC7511	ERR278413
24037	OXC7512	ERR278414
24038	OXC7513	ERR278415
24119	OXC7514	ERR278498
24120	OXC7515	ERR278499
24121	OXC7516	ERR278500
24122	OXC7517	ERR278501
24123	OXC7518	ERR278502
24124	OXC7520	ERR278503
24125	OXC7521	ERR278504
24126	OXC7522	ERR278505
24128	OXC7524	ERR278507
24129	OXC7525	ERR278508
24130	OXC7526	ERR278509
24131	OXC7527	ERR278510
24132	OXC7528	ERR278511
24133	OXC7529	ERR278512
24134	OXC7530	ERR278513
24474	OXC7531	ERR330458
24475	OXC7532	ERR330466
24476	OXC7533	ERR330474

PubMLST id	Isolate	ENA accession
24477	OXC7534	ERR330482
24479	OXC7536	ERR330498
24480	OXC7537	ERR330506
24481	OXC7538	ERR330514
24482	OXC7541	ERR330522
24483	OXC7542	ERR330530
24484	OXC7543	ERR330538
24485	OXC7544	ERR330546
24486	OXC7547	ERR330459
24487	OXC7548	ERR330467
24488	OXC7549	ERR330475
24489	OXC7551	ERR330483
24490	OXC7552	ERR330491
24491	OXC7553	ERR330499
24492	OXC7554	ERR330507
24493	OXC7556	ERR330515
24494	OXC7557	ERR330523
24495	OXC7558	ERR330531
24496	OXC7559	ERR330539
24497	OXC7560	ERR330547
24498	OXC7561	ERR330460
24499	OXC7562	ERR330468
24500	OXC7563	ERR330476
24501	OXC7564	ERR330484
24502	OXC7565	ERR330492
24503	OXC7566	ERR330500
24504	OXC7567	ERR330508
24505	OXC7568	ERR330516
24506	OXC7569	ERR330524
24507	OXC7570	ERR330532
24508	OXC7571	ERR330540
24509	OXC7572	ERR330548
24510	OXC7573	ERR330461
24512	OXC7575	ERR330477
24513	OXC7576	ERR330485
24515	OXC7578	ERR330501
24516	OXC7580	ERR330509
24517	OXC7583	ERR330517
24518	OXC7584	ERR330525
24519	OXC7585	ERR330533
24520	OXC7586	ERR330541
24521	OXC7587	ERR330549

PubMLST id	Isolate	ENA accession
24522	OXC7588	ERR330462
24523	OXC7589	ERR330470
24524	OXC7592	ERR330478
24525	OXC7593	ERR330486
24526	OXC7594	ERR330494
24527	OXC7595	ERR330502
24528	OXC7596	ERR330510
24529	OXC7597	ERR330518
24530	OXC7598	ERR330526
24531	OXC7599	ERR330534
24532	OXC7600	ERR330542
24533	OXC7601	ERR330550
24534	OXC7603	ERR330463
24535	OXC7604	ERR330471
24536	OXC7606	ERR330479
24537	OXC7607	ERR330487
24538	OXC7608	ERR330495
24539	OXC7609	ERR330503
24540	OXC7611	ERR330511
24541	OXC7612	ERR330519
24542	OXC7613	ERR330527
24543	OXC7614	ERR330535
24544	OXC7615	ERR330543
24545	OXC7616	ERR330551
24546	OXC7617	ERR330464
24547	OXC7618	ERR330472
24548	OXC7619	ERR330480
24549	OXC7620	ERR330488
24550	OXC7621	ERR330496
24551	OXC7622	ERR330504
24552	OXC7623	ERR330512
24553	OXC7624	ERR330520
24554	OXC7625	ERR330528
24555	OXC7626	ERR330536
24556	OXC7627	ERR330544
24557	OXC7628	ERR330552
24558	OXC7629	ERR330465
24559	OXC7630	ERR330473
24560	OXC7631	ERR330481
24561	OXC7632	ERR330489
24562	OXC7633	ERR330497
24563	OXC7634	ERR330505

PubMLST id	Isolate	ENA accession
24564	OXC7635	ERR330513
24565	OXC7636	ERR330521
24566	OXC7637	ERR330529
24567	OXC7638	ERR330537
24568	OXC7639	ERR330545
24569	OXC7640	ERR330553
24570	OXC7756	ERR330554
24571	OXC7759	ERR330562
24572	OXC7761	ERR330570
24573	OXC7764	ERR330578
24574	OXC7765	ERR330586
24575	OXC7766	ERR330594
24576	OXC7767	ERR330602
24577	OXC7768	ERR330610
24578	OXC7769	ERR330618
24579	OXC7770	ERR330626
24580	OXC7772	ERR330634
24581	OXC7773	ERR330642
24582	OXC7775	ERR330555
24583	OXC7776	ERR330563
24584	OXC7777	ERR330571
24585	OXC7778	ERR330579
24586	OXC7779	ERR330587
24587	OXC7780	ERR330595
24588	OXC7781	ERR330603
24589	OXC7782	ERR330611
24590	OXC7786	ERR330619
24591	OXC7788	ERR330627
24592	OXC7789	ERR330635
24593	OXC7790	ERR330643
24594	OXC7791	ERR330556
24595	OXC7792	ERR330564
24596	OXC7793	ERR330572
24597	OXC7796	ERR330580
24598	OXC7797	ERR330588
24599	OXC7798	ERR330596
24600	OXC7799	ERR330604
24601	OXC7800	ERR330612
24602	OXC7802	ERR330620
24603	OXC7803	ERR330628
24604	OXC7806	ERR330636
24605	OXC7807	ERR330644

PubMLST id	Isolate	ENA accession
24606	OXC7808	ERR330557
24607	OXC7809	ERR330565
24608	OXC7810	ERR330573
24609	OXC7811	ERR330581
24610	OXC7812	ERR330589
24611	OXC7813	ERR330597
24612	OXC7814	ERR330605
24614	OXC7816	ERR330621
24615	OXC7818	ERR330629
24616	OXC7819	ERR330637
24617	OXC7820	ERR330645
24618	OXC7821	ERR330558
24619	OXC7822	ERR330566
24620	OXC7823	ERR330574
24621	OXC7824	ERR330582
24622	OXC7825	ERR330590
24623	OXC7826	ERR330598
24624	OXC7827	ERR330606
24625	OXC7828	ERR330614
24626	OXC7829	ERR330622
24627	OXC7830	ERR330630
24629	OXC7832	ERR330646
24630	OXC7833	ERR330559
24632	OXC7835	ERR330575
24633	OXC7836	ERR330583
24636	OXC7839	ERR330607
24637	OXC7840	ERR330615
24638	OXC7841	ERR330623
24639	OXC7842	ERR330631
24640	OXC7843	ERR330639
24641	OXC7844	ERR330647
24642	OXC7845	ERR330560
24643	OXC7846	ERR330568
24644	OXC7847	ERR330576
24645	OXC7848	ERR330584
24646	OXC7849	ERR330592
24647	OXC7850	ERR330600
24648	OXC7851	ERR330608
24649	OXC7852	ERR330616
24898	OXC7641	ERR343003
24899	OXC7642	ERR343010
24900	OXC7643	ERR343017

PubMLST id	Isolate	ENA accession
24901	OXC7644	ERR343024
24903	OXC7646	ERR343040
24904	OXC7647	ERR343048
24905	OXC7648	ERR343056
24906	OXC7649	ERR343064
24907	OXC7650	ERR343072
24908	OXC7653	ERR343078
24909	OXC7654	ERR343085
24910	OXC7655	ERR343004
24911	OXC7656	ERR343011
24912	OXC7657	ERR343018
24913	OXC7658	ERR343025
24914	OXC7659	ERR343033
24915	OXC7660	ERR343041
24916	OXC7661	ERR343049
24917	OXC7662	ERR343057
24918	OXC7663	ERR343065
24919	OXC7664	ERR343073
24920	OXC7665	ERR343079
24921	OXC7666	ERR343086
24922	OXC7667	ERR343005
24923	OXC7668	ERR343012
24924	OXC7669	ERR343019
24925	OXC7670	ERR343026
24926	OXC7672	ERR343034
24927	OXC7673	ERR343042
24928	OXC7674	ERR343050
24929	OXC7675	ERR343058
24930	OXC7676	ERR343066
24931	OXC7677	ERR343074
24932	OXC7678	ERR343080
24933	OXC7679	ERR343087
24934	OXC7680	ERR343006
24935	OXC7682	ERR343013
24936	OXC7683	ERR343020
24937	OXC7686	ERR343027
24938	OXC7687	ERR343035
24939	OXC7689	ERR343043
24940	OXC7692	ERR343051
24941	OXC7693	ERR343059
24942	OXC7694	ERR343067
24943	OXC7695	ERR343075

PubMLST id	Isolate	ENA accession
24944	OXC7696	ERR343081
24945	OXC7697	ERR343088
24946	OXC7698	ERR343007
24947	OXC7699	ERR343014
24948	OXC7700	ERR343021
24949	OXC7701	ERR343028
24950	OXC7702	ERR343036
24951	OXC7704	ERR343044
24952	OXC7705	ERR343052
24953	OXC7706	ERR343060
24954	OXC7707	ERR343068
24955	OXC7709	ERR343076
24956	OXC7711	ERR343082
24957	OXC7712	ERR343089
24958	OXC7716	ERR343029
24959	OXC7717	ERR343037
24960	OXC7718	ERR343045
24961	OXC7719	ERR343053
24962	OXC7720	ERR343061
24963	OXC7721	ERR343069
24964	OXC7722	ERR343077
24965	OXC7724	ERR343083
24966	OXC7725	ERR343090
24967	OXC7726	ERR343008
24968	OXC7727	ERR343015
24969	OXC7728	ERR343022
24970	OXC7731	ERR343030
24971	OXC7732	ERR343038
24972	OXC7734	ERR343046
24973	OXC7735	ERR343054
24974	OXC7736	ERR343062
24975	OXC7738	ERR343070
24976	OXC7741	ERR343084
24977	OXC7742	ERR343091
24978	OXC7743	ERR343009
24979	OXC7744	ERR343016
24980	OXC7745	ERR343023
24981	OXC7746	ERR343031
24982	OXC7747	ERR343039
24983	OXC7748	ERR343047
24984	OXC7749	ERR343055
24985	OXC7750	ERR343063

PubMLST id	Isolate	ENA accession
24986	OXC7752	ERR343071
24987	OXC7755	ERR343092
24988	OXC7853	ERR348862
24989	OXC7854	ERR348870
24990	OXC7855	ERR348878
24991	OXC7856	ERR348886
24992	OXC7857	ERR348894
24993	OXC7858	ERR348902
24994	OXC7859	ERR348910
24995	OXC7860	ERR348918
24996	OXC7861	ERR348926
24997	OXC7862	ERR348934
24999	OXC7864	ERR348950
25000	OXC7865	ERR348863
25001	OXC7866	ERR348871
25002	OXC7867	ERR348879
25003	OXC7868	ERR348887
25004	OXC7869	ERR348895
25005	OXC7870	ERR348903
25006	OXC7871	ERR348911
25007	OXC7872	ERR348919
25008	OXC7873	ERR348927
25009	OXC7874	ERR348935
25010	OXC7875	ERR348943
25011	OXC7876	ERR348951
25012	OXC7877	ERR348864
25013	OXC7879	ERR348872
25014	OXC7881	ERR348880
25015	OXC7882	ERR348888
25016	OXC7883	ERR348896
25017	OXC7884	ERR348904
25018	OXC7887	ERR348912
25019	OXC7888	ERR348920
25020	OXC7889	ERR348928
25021	OXC7890	ERR348936
25022	OXC7891	ERR348944
25023	OXC7892	ERR348952
25025	OXC7894	ERR348873
25026	OXC7895	ERR348881
25027	OXC7896	ERR348889
25028	OXC7897	ERR348897
25030	OXC7899	ERR348913

PubMLST id	Isolate	ENA accession
25031	OXC7900	ERR348921
25032	OXC7901	ERR348929
25033	OXC7902	ERR348937
25034	OXC7903	ERR348945
25035	OXC7904	ERR348953
25037	OXC7907	ERR348882
25038	OXC7908	ERR348890
25039	OXC7909	ERR348898
25040	OXC7910	ERR348906
25041	OXC7911	ERR348914
25042	OXC7912	ERR348922
25043	OXC7913	ERR348930
25044	OXC7914	ERR348938
25045	OXC7915	ERR348946
25046	OXC7916	ERR348954
25047	OXC7917	ERR348867
25048	OXC7918	ERR348875
25049	OXC7919	ERR348883
25050	OXC7920	ERR348891
25051	OXC7922	ERR348899
25053	OXC7924	ERR348915
25054	OXC7925	ERR348923
25055	OXC7926	ERR348931
25056	OXC7927	ERR348939
25057	OXC7928	ERR348947
25058	OXC7929	ERR348955
25059	OXC7931	ERR348868
25060	OXC7932	ERR348876
25061	OXC7933	ERR348884
25063	OXC7938	ERR348900
25064	OXC7939	ERR348908
25065	OXC7940	ERR348916
25066	OXC7943	ERR348924
25067	OXC7944	ERR348932
25068	OXC7945	ERR348940
25069	OXC7906	ERR348874
25104	OXC7545	ERR348989
25105	OXC7555	ERR348997
25106	OXC7579	ERR349005
25107	OXC7582	ERR349013
25108	OXC7602	ERR349021
25109	OXC7610	ERR349029

PubMLST id	Isolate	ENA accession
25110	OXC7758	ERR349037
25111	OXC7801	ERR349045
25112	OXC7805	ERR349053
25113	OXC7878	ERR349027
25114	OXC7885	ERR349011
25115	OXC7886	ERR349019
25116	OXC7921	ERR349003
25117	OXC7930	ERR348995
25118	OXC7935	ERR348987
25119	OXC7937	ERR348979
25120	OXC7941	ERR348971
25374	OXC7954	ERR386238
25375	OXC7956	ERR386239
25376	OXC7957	ERR386240
25377	OXC7959	ERR386241
25378	OXC7960	ERR386242
25379	OXC7963	ERR386243
25380	OXC7964	ERR386244
25381	OXC7966	ERR386245
25382	OXC7967	ERR386246
25384	OXC7969	ERR386248
25385	OXC7970	ERR386249
25386	OXC7971	ERR386250
25387	OXC7972	ERR386251
25388	OXC7973	ERR386252
25389	OXC7974	ERR386253
25390	OXC7975	ERR386254
25391	OXC7976	ERR386255
25392	OXC7977	ERR386256
25393	OXC7978	ERR386257
25394	OXC7979	ERR386258
25395	OXC7981	ERR386259
25396	OXC7982	ERR386260
25397	OXC7984	ERR386261
25398	OXC7986	ERR386262
25399	OXC7987	ERR386263
25400	OXC7988	ERR386264
25401	OXC7991	ERR386265
25403	OXC7995	ERR386267
25404	OXC7997	ERR386268
25405	OXC7523	ERR494547
25406	OXC8001	ERR386270

PubMLST id	Isolate	ENA accession
25407	OXC8003	ERR386271
25408	OXC8017	ERR386272
25409	OXC8022	ERR386273
25410	OXC8024	ERR386274
25411	OXC8036	ERR386275
25412	OXC8037	ERR386276
25413	OXC8044	ERR386277
25414	OXC8056	ERR386278
25415	OXC8060	ERR386279
25416	OXC8066	ERR386280
25417	OXC8068	ERR386281
25418	OXC8069	ERR386282
25419	OXC8076	ERR386283
25420	OXC8079	ERR386284
25421	OXC8081	ERR386285
25422	OXC8083	ERR386286
25423	OXC8084	ERR386287
25424	OXC8085	ERR386288
25425	OXC8086	ERR386289
25426	OXC8089	ERR386290
25427	OXC8094	ERR386291
25428	OXC8095	ERR386292
25430	OXC8106	ERR386294
25449	OXC7343	ERR386313
25450	OXC7399	ERR386314
25452	OXC7652	ERR386316
25453	OXC7751	ERR386317
25454	OXC7757	ERR386318
25455	OXC7785	ERR483978
25456	OXC7946	ERR386320
25465	OXC7685	ERR386329
25476	OXC7947	ERR386340
25477	OXC7952	ERR386341
25478	OXC7958	ERR386342
25479	OXC7961	ERR386343
25480	OXC7962	ERR386344
25481	OXC7989	ERR386345
25482	OXC7990	ERR386346
25483	OXC7992	ERR386347
25484	OXC7994	ERR386348
25485	OXC7996	ERR386349
25486	OXC7999	ERR386350

PubMLST id	Isolate	ENA accession
25487	OXC8002	ERR386351
25488	OXC8004	ERR386352
25489	OXC8005	ERR386353
25490	OXC8006	ERR386354
25491	OXC8007	ERR386355
25492	OXC8010	ERR386356
25493	OXC8016	ERR386357
25494	OXC8019	ERR386358
25495	OXC8021	ERR386359
25496	OXC8025	ERR386360
25497	OXC8027	ERR386361
25498	OXC8029	ERR386362
25499	OXC8030	ERR386363
25500	OXC8031	ERR386364
25502	OXC8033	ERR386366
25503	OXC8035	ERR386367
25504	OXC8038	ERR386368
25505	OXC8040	ERR386369
25506	OXC8041	ERR386370
25507	OXC8042	ERR386371
25508	OXC8046	ERR386372
25509	OXC8048	ERR386373
25510	OXC8052	ERR386374
25511	OXC8058	ERR386375
25512	OXC8062	ERR386376
25513	OXC8063	ERR386377
25514	OXC8064	ERR386378
25515	OXC8065	ERR386379
25516	OXC8070	ERR386380
25517	OXC8077	ERR386381
25518	OXC8078	ERR386382
25519	OXC8080	ERR386383
25520	OXC8082	ERR386384
25521	OXC8087	ERR386385
25522	OXC8088	ERR386386
25523	OXC8090	ERR386387
25524	OXC8091	ERR386388
25525	OXC8096	ERR386389
25526	OXC8098	ERR386390
25527	OXC8099	ERR386391
25528	OXC8100	ERR386392
25529	OXC8101	ERR386393

PubMLST id	Isolate	ENA accession
25530	OXC8103	ERR386394
25537	OXC7710	ERR386401
25538	OXC7730	ERR386402
25552	OXC7318	ERR386416
25555	OXC7651	ERR386419
25567	OXC7936	ERR386431
25568	OXC7942	ERR386432
25569	OXC7949	ERR386433
25570	OXC7950	ERR386434
25571	OXC7951	ERR386435
25572	OXC7953	ERR386436
25573	OXC7955	ERR386437
25574	OXC7965	ERR386438
25575	OXC7980	ERR386439
25576	OXC7983	ERR386440
25577	OXC7985	ERR386441
25578	OXC8000	ERR386442
25579	OXC8008	ERR386443
25580	OXC8009	ERR386444
25581	OXC8011	ERR386445
25582	OXC8012	ERR386446
25583	OXC8013	ERR386447
25584	OXC8014	ERR386448
25585	OXC8015	ERR386449
25586	OXC8018	ERR386450
25587	OXC8020	ERR386451
25588	OXC8023	ERR386452
25589	OXC8026	ERR386453
25590	OXC8028	ERR386454
25591	OXC8034	ERR386455
25592	OXC8039	ERR386456
25593	OXC8043	ERR386457
25595	OXC8049	ERR386459
25596	OXC8050	ERR386460
25598	OXC8053	ERR386462
25600	OXC8055	ERR386464
25601	OXC8057	ERR386465
25602	OXC8059	ERR386466
25603	OXC8061	ERR386467
25604	OXC8067	ERR386468
25605	OXC8071	ERR386469
25606	OXC8072	ERR386470

PubMLST id	Isolate	ENA accession
25607	OXC8073	ERR386471
25608	OXC8074	ERR386472
25609	OXC8075	ERR386473
25610	OXC8092	ERR386474
25611	OXC8093	ERR386475
25612	OXC8097	ERR386476
25620	OXC7426	ERR386484
25621	OXC7434	ERR386485
25622	OXC7435	ERR386486
25623	OXC7460	ERR386487
25624	OXC7483	ERR386488
25626	OXC7550	ERR386490
25627	OXC7688	ERR386491
25628	OXC7691	ERR386492
25629	OXC7708	ERR386493
25630	OXC7729	ERR386494
25650	OXC7671	ERR386514
25651	OXC7739	ERR386515
25652	OXC7948	ERR386516
25654	OXC7783	ERR386518
27865	OXC7713	ERR351536
27866	OXC7714	ERR351537
27867	OXC7715	ERR351538
27868	OXC7740	ERR351539
27869	OXC7753	ERR351540
27870	OXC7754	ERR351541
27877	OXC7352	ERR459473
27878	OXC7681	ERR459474
27879	OXC7723	ERR459476
27880	OXC7737	ERR459477
27881	OXC7771	ERR459478
27882	OXC7787	ERR459479
27883	OXC7794	ERR459480
27884	OXC7804	ERR459481
27885	OXC7817	ERR459482
28559	OXC8051	ERR483981

Table S2 Details of isolates used to confirm membership of *C. coli* clades 1, 2, and 3

PubMLST id	Isolate	Clade	Reference
2473	RM4661	1	-
24266	8808	2	(1)
24267	2544	2	(1)
24268	6873	2	(1)
24269	dfvf1656	2	(1)
24270	FSA05.280042	3	(1)
24271	8096	3	(1)
24272	dfvf1912	3	(1)
24273	4944	3	(1)
24274	RM4931	3	(1)
32046	FB1	1	-
32237	CVM N29710	1	(2)
32255	RM1875	1	-
32289	RM5611	1	-
32298	15-537360	1	(3)

Table S3 RT-PCR cycle thresholds for *mapA* and *ceuE* primer and probe combinations[a]

Gene	Primer and probe allele (n^a)	C_T^b Maximum	Minimum	Average	Polymorphisms (n)[c]
mapA	1 (68)	22	12	15	0-0-0
	2 (11)	20	15	17	2-0-0
	3 (3)	19	15	17	0-1-0
	4 (3)	17	16	16	1-0-2
	5 (4)	17	15	16	0-1-1
	6 (1)	-	-	14	1-1-0
	7 (2)	27	25	26	3-0-2
	8 (1)	-	-	12	0-0-1
	9 (2)	21	17	19	2-1-0
	10 (1)	-	-	14	0-1-0
	11 (1)	-	-	19	2-0-2
	12 (1)	-	-	14	0-1-0
	13 (1)	-	-	15	1-2-0
	14 (1)	-	-	15	0-1-0
	15 (9)	N/D[d]	N/D	N/D	4-6-6
	16 (3)	N/D	N/D	N/D	4-7-6
	17 (3)	N/D	N/D	N/D	3-6-6
	18 (2)	N/D	N/D	N/D	4-6-7
	19 (1)	N/D	N/D	N/D	4-6-8
	20 (1)	N/D	N/D	N/D	2-6-7
	21 (1)	N/D	N/D	N/D	4-7-7
	22 (1)	N/D	N/D	N/D	0-5-6
	23 (1)	-	-	33.29	0-0-6
ceuE	1 (18)	N/D	N/D	N/D	4-3-5
	2 (29)	N/D	N/D	N/D	4-3-4
	3 (8)	N/D	N/D	N/D	4-3-5

4 (7)	N/D	N/D	N/D	3-3-5
5 (12)	N/D	N/D	N/D	3-3-5
6 (1)	N/D	N/D	N/D	4-3-5
7 (1)	N/D	N/D	N/D	4-3-4
8 (3)	N/D	N/D	N/D	4-3-5
9 (1)	N/D	N/D	N/D	4-3-5
10 (1)	N/D	N/D	N/D	3-2-5
11 (1)	N/D	N/D	N/D	4-3-5
12 (2)	N/D	N/D	N/D	3-3-5
13 (1)	N/D	N/D	N/D	4-3-6
14 (1)	N/D	N/D	N/D	4-3-3
15 (1)	N/D	N/D	N/D	4-4-5
16 (2)	N/D	N/D	N/D	4-3-5
17 (2)	N/D	N/D	N/D	3-3-5
18 (1)	N/D	N/D	N/D	4-3-4
19 (1)	N/D	N/D	N/D	4-3-4
20 (1)	N/D	N/D	N/D	4-4-4
21 (1)	N/D	N/D	N/D	4-3-5
22 (19)	22	12	15	0-0-0
23 (1)	-	-	13	1-0-0
24 (1)	-	-	6	1-0-0
25 (1)	-	-	37	5-2-1
26 (1)	-	-	32	4-2-1

[a] Number of representative isolates tested.

[b] C_T, cycle threshold.

[c] Number of polymorphisms in [forward primer - probe - reverse primer].

[d] N/D, not detected.

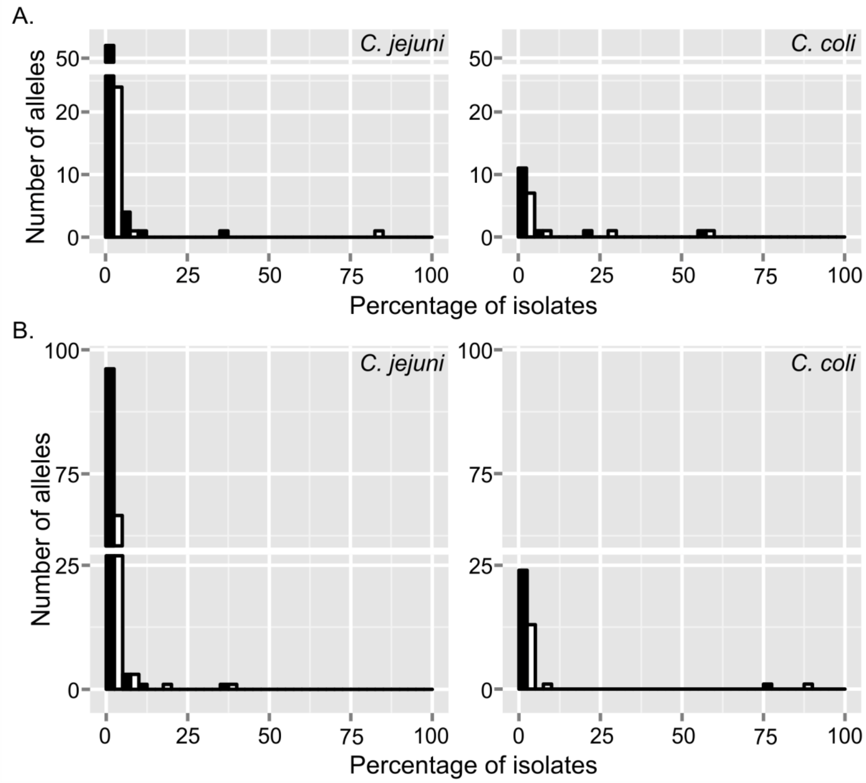

Fig. S1 Frequency distribution of *mapA* (A) and *ceuE* (B) nucleotide (black) and protein alleles (white) in *C. jejuni* and *C. coli*.

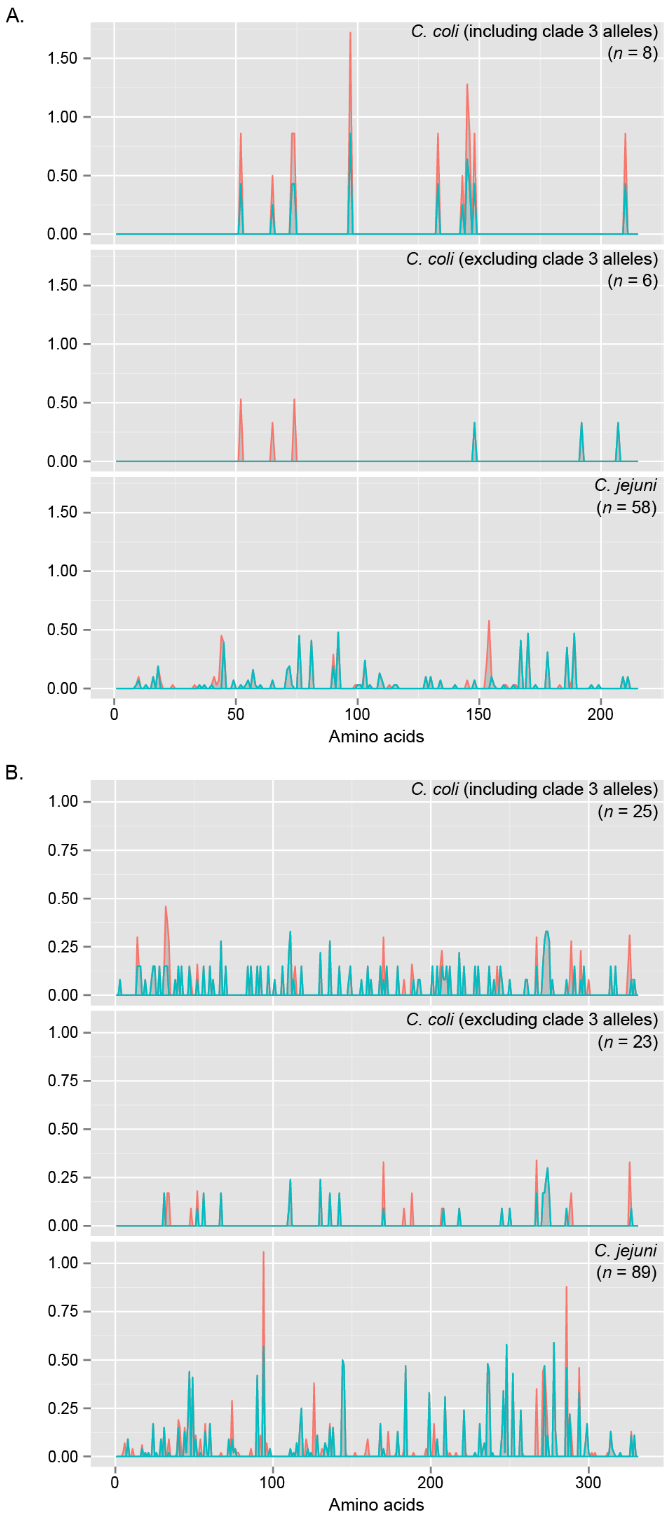

Fig. S2 Distribution of synonymous and non-synonymous substitutions across *mapA* (A) and *ceuE* (B) in *C. jejuni* and *C. coli* as determined using SNAP (www.hiv.lanl.gov) (4). The *y*-axis indicates the average rate of synonymous (blue) and non-synonymous (red) substitutions per codon across all pairwise comparisons. Sites under positive selection were defined as those with $d_N > d_S$.

References

1. **Sheppard SK, Didelot X, Jolley KA, Darling AE, Pascoe B, Meric G, Kelly DJ, Cody A, Colles FM, Strachan NJ, Ogden ID, Forbes K, French NP, Carter P, Miller WG, McCarthy ND, Owen R, Litrup E, Egholm M, Affourtit JP, Bentley SD, Parkhill J, Maiden MC, Falush D.** 2013. Progressive genome-wide introgression in agricultural *Campylobacter coli*. Molecular Ecology **22:**1051-1064.
2. **Chen Y, Mukherjee S, Hoffmann M, Kotewicz ML, Young S, Abbott J, Luo Y, Davidson MK, Allard M, McDermott P, Zhao S.** 2013. Whole-genome sequencing of gentamicin-resistant *Campylobacter coli* isolated from U.S. retail meats reveals novel plasmid-mediated aminoglycoside resistance genes. Antimicrobial Agents and Chemotherapy **57:**5398-5405.
3. **Pearson BM, Rokney A, Crossman LC, Miller WG, Wain J, van Vliet AH.** 2013. Complete Genome Sequence of the *Campylobacter coli* Clinical Isolate 15-537360. Genome Announcements **1:**e01056-01013.
4. **Korber B.** 2000. HIV signature and sequence variation analysis, p 55-72. *In* Rodrigo AG, Learn GH (ed), Computational Analysis of HIV Molecular Sequences. Kluwer Academic Publishers, Dordrecht, Netherlands.
